# Interferon-Induced Transmembrane Protein 3 Shapes an Inflamed Tumor Microenvironment and Identifies Immuno-Hot Tumors

**DOI:** 10.3389/fimmu.2021.704965

**Published:** 2021-08-11

**Authors:** Yun Cai, Wenfei Ji, Chuan Sun, Rui Xu, Xuechun Chen, Yifan Deng, Jiadong Pan, Jiayue Yang, Hongjun Zhu, Jie Mei

**Affiliations:** ^1^Department of Oncology, Nantong Third People’s Hospital Affiliated to Nantong University, Nantong, China; ^2^Department of Geriatrics, Key Lab of Geriatrics & Geriatrics Institute of Zhejiang Province, Zhejiang Hospital, Hangzhou, China; ^3^Wuxi College of Clinical Medicine, Nanjing Medical University, Nanjing, China; ^4^College of Pediatrics, Nanjing Medical University, Nanjing, China; ^5^Department of Endocrinology, Wuxi People’s Hospital Affiliated to Nanjing Medical University, Wuxi, China

**Keywords:** IFITM3, tumor immunity, pancancer, bioinformatics analysis, immunotherapy

## Abstract

Interferon-induced transmembrane protein 3 (IFITM3) is an interferon-induced membrane protein, which has been identified as a functional gene in multiple human cancers. The role of IFITM3 in cancer has been preliminarily summarized, but its relationship to antitumor immunity is still unclear. A pancancer analysis was conducted to investigate the expression pattern and immunological role of IFITM3 based on transcriptomic data downloaded from The Cancer Genome Atlas (TCGA) database. Next, correlations between IFITM3 and immunological features in the bladder cancer (BLCA) tumor microenvironment (TME) were assessed. In addition, the role of IFITM3 in estimating the clinical characteristics and the response to various therapies in BLCA was also evaluated. These results were next confirmed in the IMvigor210 cohort and a recruited cohort. In addition, correlations between IFITM3 and emerging immunobiomarkers, such as microbiota and N6-methyladenosine (m6A) genes, were assessed. IFITM3 was enhanced in most tumor tissues in comparison with adjacent tissues. IFITM3 was positively correlated with immunomodulators, tumor-infiltrating immune cells (TIICs), cancer immunity cycles, and inhibitory immune checkpoints. In addition, IFITM3 was associated with an inflamed phenotype and several established molecular subtypes. IFITM3 expression also predicted a notably higher response to chemotherapy, anti-EGFR therapy, and immunotherapy but a low response to anti-ERBB2, anti-ERBB4, and antiangiogenic therapy. In addition, IFITM3 was correlated with immune-related microbiota and m6A genes. In addition to BLCA, IFITM3 is expected to be a marker of high immunogenicity in most human cancers. In conclusion, IFITM3 expression can be used to identify immuno-hot tumors in most cancers, and IFITM3 may be a promising pancancer biomarker to estimate the immunological features of tumors.

## Introduction

IFITM3 is an interferon-induced membrane protein that contributes to conferring immunity to influenza A H1N1 virus, West Nile virus, and dengue virus (1). In general, IFITM3-mediated antiviral activity primarily manifests at the entry stage (2). CD225 domain is one of most important domains in IFITM3 protein, which is a critical part of its antiviral properties by direct inhibition of membrane fusion (3, 4). Emerging evidence suggests that IFITM3 regulates endocytic trafficking in the absence of infection as well. Spence et al. reported that IFITM3 promotes the degradation of EGFR in lysosomes under EGF stimulation (5). However, it is unclear whether this effect mediates the oncogenesis and progression of cancers.

Over the last few decades, IFITM3 has been identified as a functional gene in multiple human cancers, indirectly affecting ongoing inflammation and immune and cancer epithelial-to-mesenchymal transition (EMT)-associated pathways (6). In hepatocellular carcinoma, a novel pathway of the IFITM3-ERK1/2-c-myc regulatory loop has been identified, which contributes to tumor progression (7). In addition, IFITM3 levels respond to the stimulation of TGF-β in glioma, and the knockdown of IFITM3 blocked TGF-β-mediated invasion of tumor cells by suppressing STAT3 phosphorylation (8). Moreover, mTOR inhibitor (Rapamycin) treatment results in downregulation of IFITM3 (9), indicating that inhibition of mTOR signaling is a promising strategy to inhibit IFITM3-mediated cancer progression. Logically, considering that IFITM3 is mediated by interferon and regulates the phosphorylation of STAT3, we speculate that IFITM3 might be essential for antitumor immunity. However, the relationship between IFITM3 and the landscape of tumor immunity has not been explored.

Tumor tissue consists of not only tumor cells but also immune cells, stromal cells, vascular networks, and many other cellular and noncellular components, which contribute to the formation of the tumor microenvironment (TME) (10). The high heterogeneity within the TME remains an inexorable obstacle for the understanding of cancer (10). Based on the characteristics of the TME, tumors can be divided into hot and cold tumors. Hot tumors are characterized by T-cell infiltration and molecular characteristics of immune activation, whereas cold tumors exhibit striking features of T-cell absence or exclusion (10). In principle, hot tumors exhibit higher response rates to immunotherapy, such as anti-PD-1/PD-1 therapy (11). Thus, evaluating tumor immunogenicity using potential biomarkers is essential for the identification of patient populations who are sensitive to immunotherapy.

In this study, a pancancer analysis of the expression and immunological characteristics of IFITM3 was first performed, and the results revealed that IFITM3 was highly correlated with immunological factors in most cancers, but the tightest correlation was found in bladder cancer (BLCA). In addition, high IFITM3 expression could be used to identify an inflamed TME and immuno-hot tumors in BLCA, and IFITM3 had the potential to predict the molecular subtype and therapeutic efficacy of various therapies in BLCA. Moreover, we further expanded and validated the immunological role of IFITM3 in multiple cancers. Taken together, IFITM3 is a pancancer biomarker for identifying immunogenicity in human cancers.

## Materials and Methods

### Data Source and Preprocessing

The pancancer normalized gene expression profiles, 450K methylation data, and clinical annotations of The Cancer Genome Atlas (TCGA) datasets were obtained from the online data portal UCSC Xena (https://xenabrowser.net/datapages/). In addition, the copy number variant (CNV) information processed by the GISTIC algorithm was also acquired from UCSC Xena. The somatic mutation data were retrieved from TCGA and then preprocessed in the R package “maftools”. The abbreviations for various cancer types are exhibited in **Supplementary Table S1**.

In addition, the immunotherapeutic advanced urothelial cancer cohort (IMvigor210 cohort) was included in our study (12). Based on the Creative Commons 3.0 License, the complete expression data and clinical annotations could be downloaded from http://research-pub.gene.com/IMvigor210CoreBiologies/. Moreover, three hypoxia scores available in TCGA (Buffa, Ragnum, and Winter hypoxia scores) were obtained from the ciboPortal website (http://www.cbioportal.org/), which represent the activity of hypoxia-related pathways developed by different research groups (13).

### Oncomine Database Analysis

The Oncomine database (https://www.oncomine.org/) was applied to assess the transcriptional expression level of IFITM3 in tumor and normal tissues (14). Regarding the differential analysis of IFITM3 between tumor and normal samples, the thresholds were set as follows: analysis type, cancer vs. normal; threshold *p*-value, 0.05; threshold fold change, all; threshold gene rank, top 10%; and data type, mRNA.

### Linked Omics Database Analysis

The Linked Omics database (http://www.linkedomics.org/login.php) is a web-based tool to analyze multidimensional datasets (15). The functional roles of IFITM3 in BLCA were predicted using the Linked Omics tool in terms of Gene Ontology (GO) and Kyoto Encyclopedia of Genes and Genomes (KEGG) analyses by gene set enrichment analysis (GSEA). Default options were used for all parameters.

### Estimation of the Immunological Characteristics of the TME

Considering that the bulk transcriptomic data from patients included both immune and tumor cells, we estimated the immunological characteristics of the TME of each patient. Information on 122 immunomodulators, well-known effector genes of tumor-infiltrating immune cells (TIICs), and 18 specific genes associated with T-cell inflammation and their weighting coefficients was collected from previous studies (16, 17). The ESTIMATE algorithm, a method that uses gene expression signatures to infer the fraction of stromal and immune cells in tumor samples, was used to assess Tumor Purity, ESTIMATE Score, Immune Score, and Stromal Score. Yoshihara et al. defined ssGSEA based on the signatures related to stromal tissue and immune cell infiltration as Stromal and Immune scores and combined the stromal and immune scores as the ESTIMATE score, and Tumor Purity was defined as the relative proportion of tumor cells in a tumor tissue (18). Moreover, to avoid the miscalculation caused by various algorithms when estimating the levels of TIICs, we comprehensively computed the relative abundance of TIICs using the following independent algorithms: TIMER (19), EPIC (20), MCP-counter (21), quanTIseq (22), and TISIDB (23). Because each step of the cancer immune cycle plays a significant role in reflecting the anticancer immune response and determining the fate of tumor cells, we next estimated the activities of each step by single sample gene set enrichment analysis (ssGSEA) according to the expression level of specific signatures of each step (24).

To confirm the role of IFITM3 in mediating cancer immunity in BLCA, we divided the patients into high- and low-IFITM3 groups with a 50% cutoff according to the transcriptional levels of IFITM3 and then compared the difference in immunological features of the TME between the two groups.

### Calculation of the Enrichment Scores of Various Gene Signatures

We analyzed the oncogenic pathways that were associated with an inflamed TME, targeted therapy, and immunotherapy responses according to previous research (25). The enrichment scores of these signatures were calculated using the R package “GSVA” (26).

### Prediction of Chemotherapeutic Response

The role of IFITM3 in predicting the response to chemotherapy was also assessed. The BLCA-related drug-target genes were extracted using the DrugBank database, and the difference in their expression in the high- and low-IFITM3 groups was compared. To further predict the response to several common chemotherapeutic drugs for each patient, the prediction process was performed by the R package “pRRophetic” based on the Cancer Genome Project (CGP) database (https://www.sciencedirect.com/topics/neuroscience/cancer-genome-project). In the prediction process, the half-maximal inhibitory concentration (IC_50_) of the sample was estimated by ridge regression, and the prediction accuracy was evaluated by 10-fold cross-validation based on the CGP training set. Default options were used for all parameters (27). Next, it was notable that the mutations of several crucial genes, including TP53, RB1, ATM, ERBB2, ERCC2, and FANCC, were indicators of the response to chemotherapy in BLCA (25, 28). Thus, we compared the mutation rates of these genes in the high- and low-IFITM3 groups.

### Estimation of the Molecular Subtypes in BLCA

There are several molecular subtype systems, including the CIT, Lund, MDA, TCGA, Baylor, UNC, and consensus subtypes (29–35). The subtype information in the TCGA database was obtained from previous research (25). The subtype information in the IMvigor210 cohort (including Lund and TCGA subtypes) was downloaded from the official website. Gene markers involved in 12 BLCA signatures that are peculiar to different molecular subtypes were obtained from previous research (25), and these signatures were performed by the Bladder Cancer Molecular Taxonomy Group (29). Next, we evaluated the association between IFITM3 and various molecular subtypes and BLCA gene signatures.

### Identification of Immune-Related m6A Genes

According to a recent publication (36), we identified 64 m6A regulators and m6A interactive protein-coding genes with a high coexpression tendency and selected the genes whose absolute Pearson correlation with the T-cell inflamed score was ≥0.3 or ≤−0.3 and had a *p*-value ≤ 0.05.

### Discovery of Immune-Related Tumor Microorganism

To explore microorganisms associated with antitumor immunity in BLCA, we downloaded the relative abundance of ~1,400 microorganisms from the online data portal ciboPortal (http://www.cbioportal.org/) and selected microorganisms whose absolute Pearson correlation with the T-cell inflamed score was ≥0.3 or ≤−0.3 and had a *p*-value ≤ 0.05.

### Clinical Samples

The BLCA tissue microarray (TMA, HBlaU079Su01) was purchased from Outdo Biotech (Shanghai, China). The HBlaU079Su01 microarray contained 63 BLCA and 16 adjacent samples. Ethical approval for the study of tissue microarray slides was granted by the Clinical Research Ethics Committee, Outdo Biotech (Shanghai, China).

### Immunohistochemistry

Immunohistochemistry (IHC) staining was directly conducted on the HBlaU079Su01 TMA with standard procedures. The primary antibodies used were as follows: anti−IFITM3 (1:3000 dilution, Cat. ab109429, Abcam, Cambridge, UK), anti−PD-L1 (Ready-to-use, Cat. GT2280, GeneTech, Shanghai, China), and anti−CD8 (Ready-to-use, Cat. PA067, Abcarta, Suzhou, China). Antibody staining was visualized with DAB and hematoxylin counterstain, and stained sections were scanned using Aperio Digital Pathology Slide Scanners.

### Semiquantitative Scoring

The stained TMA was independently assessed by two pathologists. For semiquantitative assessment of IFITM3 and PD-L1 expression only on tumor cells, the percentage of positively stained cells was scored as 0–4: 0 (<1%), 1 (1–5%), 2 (6–25%), 3 (26–50%) and 4 (>50%). The staining intensity was scored as 0–3: 0 (negative), 1 (weak), 2 (moderate), and 3 (strong). The immunoreactivity score (IRS) equals the percentage of positive cells multiplied by the staining intensity. For semiquantitative assessment of CD8 staining, the infiltration level was assessed by estimating the percentage of cells with strong intensity of membrane staining in total stromal cells. Tumors were divided into three phenotypes according to the spatial distribution of CD8+ T cells, including the inflamed, excluded, and deserted phenotypes (25).

### Statistical Analysis

All statistical analyses shown in the figures were applied by R version 3.6.0. The significant difference in continuous variables between the two groups was measured by the Wilcoxon rank sum test, while categorical variables were compared using the chi-square test. Prognostic values of categorical variables were assessed using the log-rank test. For all analyses, a two-paired *p*-value ≤ 0.05 was considered statistically significant if not noted. Statistical significance was defined as *p-value ≤ 0.05, **p-value ≤ 0.01, ***p-value ≤ 0.001, and ****p-value ≤ 0.0001.

## Results

### Expression Pattern and Immunological Role of IFITM3 in Pancancer

First, a comprehensive analysis of the expression and prognostic value of IFITM3 in pancancer was conducted. In the Oncomine database, we discovered that IFITM3 was highly expressed in several cancers but expressed at low levels in BLCA and prostate cancer. However, the expression status of IFITM3 was inconsistent in most cancers (**Supplementary Figure S1A**). In the TCGA database, most types of cancer expressed higher IFITM3, while in CHOL, MESO, and several other cancers, IFITM3 exhibited low expression (**Supplementary Figure S1B**). Next, a pancancer survival analysis of overall survival (OS) and progression-free survival (PFS) was conducted by the Kaplan–Meier method. The prognostic value of IFITM3 in human cancers was limited. In KIRC and glioma, high expression of IFITM3 was related to a better prognosis, while in SKCM, IFITM3 was a risk prognostic factor (**Supplementary Figures S1C, D**).

**Figure 1:**
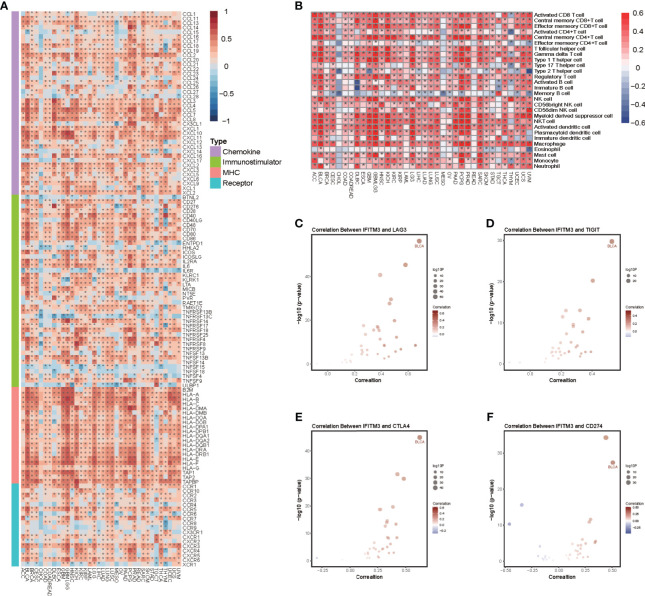
Pancancer analysis of the effect of IFITM3 on immunological status. **(A)** Correlations between IFITM3 and 122 immunomodulators (chemokines, immunostimulators, MHC, and receptors). The color indicates the correlation coefficient. The asterisks indicate significant differences assessed by Pearson analysis. **(B)** Correlations between IFITM3 and 28 TIICs calculated with the ssGSEA algorithm. The color indicates the correlation coefficient. The asterisks indicate significant differences assessed by Pearson analysis. Correlation between IFITM3 and 4 immune checkpoints, **(C)** LAG3, **(D)** TIGIT, **(E)** CTLA4, and **(F)** CD274. The dots represent cancer types. The *y*-axis represents the Pearson correlation coefficient, while the *x*-axis represents ×log_10_ (*p*-value).

We next performed a pancancer analysis to examine the immunological features of IFITM3 in all accessible tumor types in the TCGA database. The results revealed that IFITM3 exhibited positive correlations with a majority of immunomodulators in almost all cancer types (**Figure 1A**). We next calculated the infiltrating levels of TIICs in the TME using the ssGSEA algorithm. Similarly, except for CHOL and MESO, IFITM3 expression was highly correlated with most types of TIICs in multiple cancers (**Figure 1B**). In addition, we also assessed the correlations between IFITM3 and the expression of immune checkpoints, including LAG3, TIGIT, CTLA4, CD274, PDCD1, HAVCR2, CD80, and CD86, across cancers. The results showed that IFITM3 was positively related to these immune checkpoints in the pancancer pattern, and the highest correlation was observed in BLCA (**Figures 1C–F**, **Supplementary Figures S2A–D**). Collectively, these results reveal the potential of IFITM3 as an immune-related biomarker in human cancers, especially BLCA.

### Functions of IFITM3 and Regulatory Factors of Its Expression in BLCA

The functions of IFITM3 in BLCA were analyzed using the LinkedOmics tool. GO enrichment analysis predicted the functional roles of IFITM3 in terms of three categories, including biological processes, cellular components, and molecular functions. Many statistically significant terms were identified, and the top five terms of each analysis were obtained. As shown in **Supplementary Table S2**, the most critical terms were associated with immune-related processes. In addition, the regulatory factors of IFITM3 expression were also analyzed. There was no mutation in the IFITM3 gene in BLCA, indicating that gene mutation was not a dominating factor in its expression regulation (**Supplementary Figure S3A**). However, copy number amplification of IFITM3 upregulated its expression, while deletion downregulated its expression (**Supplementary Figure S3B**). Furthermore, the methylation levels of multiple sites were positively or negatively correlated with its expression (**Supplementary Figure S3C**). Overall, these results indicate that copy number alterations and methylation modifications were significant for the regulation of IFITM3 expression.

### IFITM3 Shapes an Inflamed TME in BLCA

**Figure 2:**
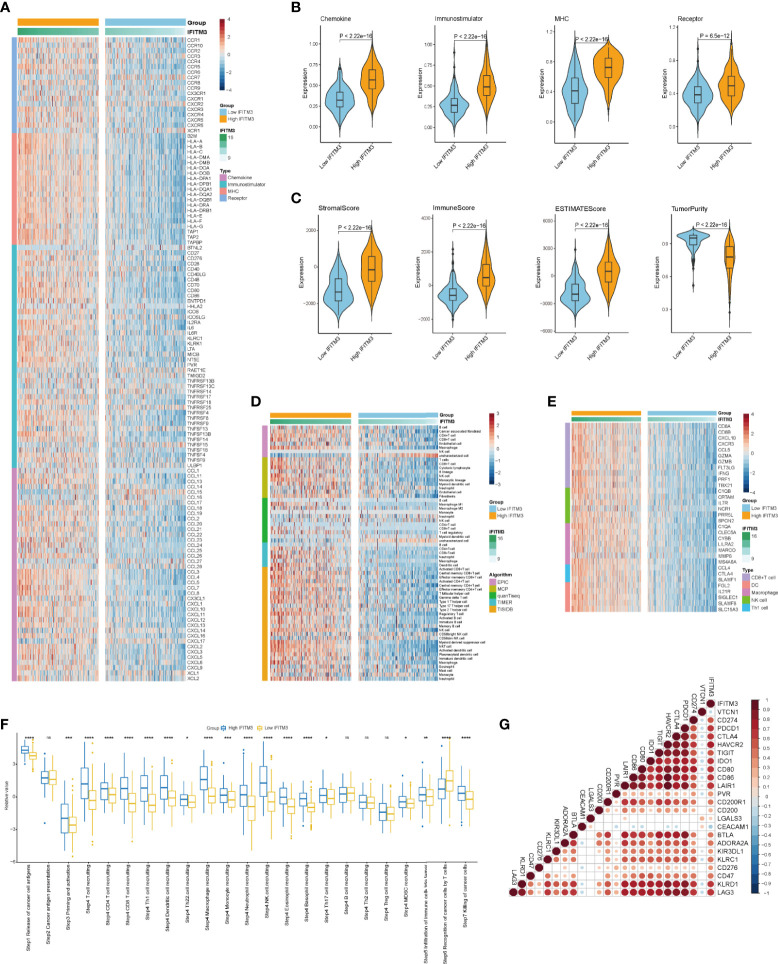
IFITM3 shapes an inflamed TME in BLCA. **(A, B)** Expression levels of 122 immunomodulators (chemokines, immunostimulators, MHC, and receptors) in the high- and low-IFITM3 groups in BLCA. **(C)** Distribution of tumor purity, ESTIMATE score, immune score, and stromal score calculated using the ESTIMATE algorithm in the high- and low-IFITM3 groups. **(D)** The levels of TIICs calculated using five algorithms (TIMER, EPIC, MCP-counter, quanTIseq, and TISIDB) in the high- and low-IFITM3 groups. **(E)** Expression levels of the gene markers of the common TIICs in the high- and low-IFITM3 groups. **(F)** The activities of the various steps of the cancer immunity cycle calculated by ssGSEA algorithm in the high- and low-IFITM3 groups. **(G)** Correlations between IFITM3 and common inhibitory immune checkpoints. The color and the values indicate the Pearson correlation coefficient. *p-value ≤ 0.05, **p-value ≤ 0.01, ***p-value ≤ 0.001, and ****p-value ≤ 0.0001. NS, no statistical significance.

Given that the highest correlation between IFITM3 and immunofactors was observed in BLCA, we subsequently explored the immunological role of IFITM3 in BLCA in the TCGA and IMvigor210 cohorts. Many chemokines, paired receptors, MHC molecules, and immunomodulators were upregulated in the high-IFITM3 group (**Figures 2A, B**, **Supplementary Figures S4A, B**). These chemokines and receptors recruit effector TIICs, including CD8+ T cells, macrophages, and antigen-presenting cells. Next, the ESTIMATE method was used to assess tumor purity, ESTIMATE score, immune score, and stromal score. Compared with the low-IFITM3 group, the high-IFITM3 group exhibited a higher ESTIMATE Score, Immune Score, and Stromal Score but lower tumor purity (**Figure 2C**, **Supplementary Figure S4C**), indicating that tumors with high IFITM3 expression were accompanied by increased immune cell infiltration. Subsequently, we estimated the infiltration levels of TIICs using five independent strategies. By using various algorithms, we found that the infiltration levels of most immune cells were remarkably upregulated in the IFITM3 group (**Figure 2D**, **Supplementary Figure S4D**). We also assessed the gene markers of common immune cells and discovered that these markers were upregulated in the high-IFITM3 group (**Figure 2E**, **Supplementary Figure S4E**). In addition, the activities associated with the cancer immunity cycle involve direct systematic performances by chemokines and other immunomodulators. In the high-IFITM3 group, the activities associated with the majority of the steps in the cycle were notably upregulated (**Figure 2F**, **Supplementary Figure S4F**). Inhibitory immune checkpoints, such as PD-1/PD-L1, were revealed to be highly expressed in the inflamed TME (37). As expected, IFITM3 was found to be highly correlated with most immune checkpoints in BLCA, including CD274, PDCD1, and CTLA4 (**Figure 2G**, **Supplementary Figure S4G**). In summary, IFITM3 is highly correlated with the inflamed TME, which may have diagnostic value in identifying the immunogenicity of BLCA.

### IFITM3 Predicts Immune Phenotype in BLCA

**Figure 3:**
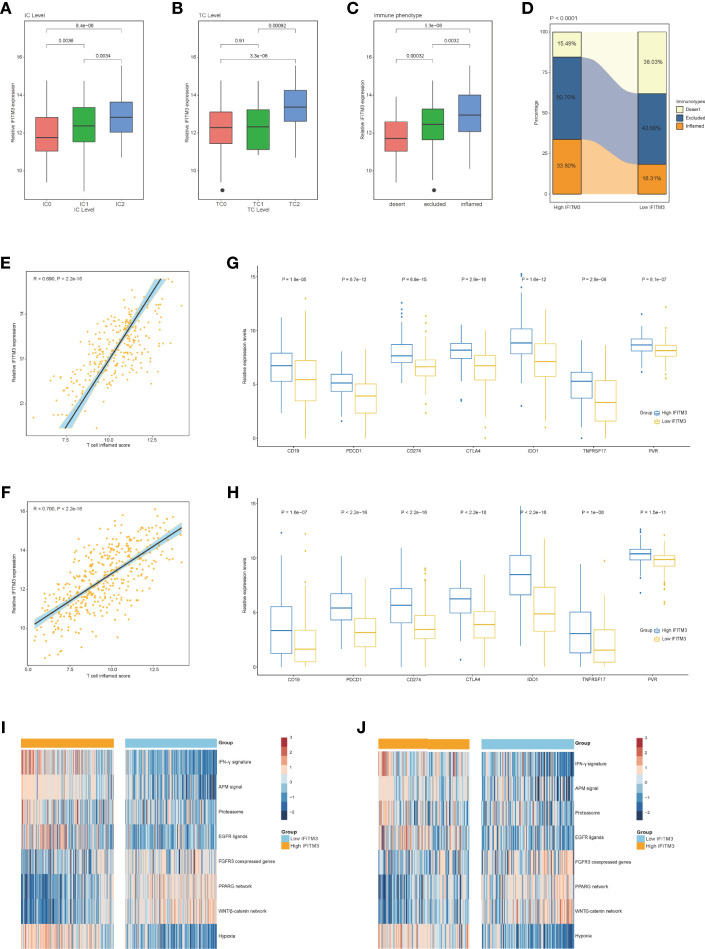
Correlation between IFITM3 and the immune phenotype in BLCA. **(A, B)** Differences in PD-L1 expression in tumor cells and immune cells between the high- and low-IFITM3 groups in the IMvigor210 cohort. **(C)** Expression of IFITM3 in all three phenotypes in the IMvigor210 cohort. **(D)** The proportions of the three phenotypes in the high- and low-IFITM3 groups in the IMvigor210 cohort. **(E, F)** Levels of T cell inflamed scores in the high- and low-IFITM3 groups in the TCGA and IMvigor210 cohorts. **(G, H)** Expression levels of immune-related targets in the high- and low-IFITM3 groups in the TCGA and IMvigor210 cohorts. **(I, J)** Enrichment scores of several immune-related signatures calculated using the “GSVA” R package in the high- and low-IFITM3 groups in the TCGA and IMvigor210 cohorts.

Logically, patients with high IFITM3 expression should exhibit a better response to immunotherapy because IFITM3 is associated with an inflamed TME. Considering that the IMvigor210 cohort contained information on PD-L1 expression in immune cells (ICs) and tumor cells (TCs), immunotypes of tumors, and response to immunotherapy, we next assessed the association between IFITM3 expression and these data. In the IMvigor210 cohort, IFITM3 exhibited the highest expression in IC2 (immune cells with the highest PD-L1 values), TC2 (tumor cells with the highest PD-L1 values), and the inflamed phenotype (**Figures 3A–C**). In addition, in the high-IFITM3 group, the proportion of the inflamed phenotype was higher, while the proportion of the deserted phenotype was lower than that in the low-IFITM3 group (**Figure 3D**). The T-cell inflamed score is established using IFN-γ-related mRNA profiles to use as an alternative for estimating the clinical response to anti-PD-1 therapy (17). In BLCA, IFITM3 expression was positively related to the T-cell inflamed score in the TCGA and IMvigor210 cohorts (**Figures 3E, F**). The expression levels of immunotargets are usually correlated with the response to immunotherapy. Encouragingly, the expression levels of frequently studied immunotargets, such as CD19, PDCD1, and CD274, were notably enhanced in the high-IFITM3 group (**Figures 3G, H**). In addition, IFITM3 positively correlated with the enrichment scores of most immunotherapy-positive gene signatures in the TCGA and IMvigor210 cohorts (**Figures 3I, J**). Considering the tight association between IFITM3 and hypoxia, we also compared the hypoxia scores in the low- and high-IFITM3 groups. The results showed that Buffa, Winter, and Ragnum hypoxia scores were higher in the high-IFITM3 group (**Supplementary Figures S5A–C**). We next assessed the association between IFITM3 expression and response to immunotherapy in the IMvigor210 cohort. However, there was no difference in the expression of IFITM3 in the CR/PR and SD/PD groups (**Supplementary Figure S6A**). We also compared the expression levels of reliable immune checkpoints in these groups. Only CD274 exhibited limited differential expression, and other molecules, such as CTLA4, PDCD1, PVR, and TIGIT, were not differentially expressed (**Supplementary Figure S6B–F**). Thus, we speculated that the response to immunotherapy depended on multiple aspects, and single gene expression analysis may have a limited value for identifying the efficacy. Regardless, IFITM3 was associated with the expression of immune checkpoints and immune phenotypes, at least in BLCA.

### IFITM3 Predicts Molecular Subtypes and Therapeutic Opportunities in BLCA

**Figure 4:**
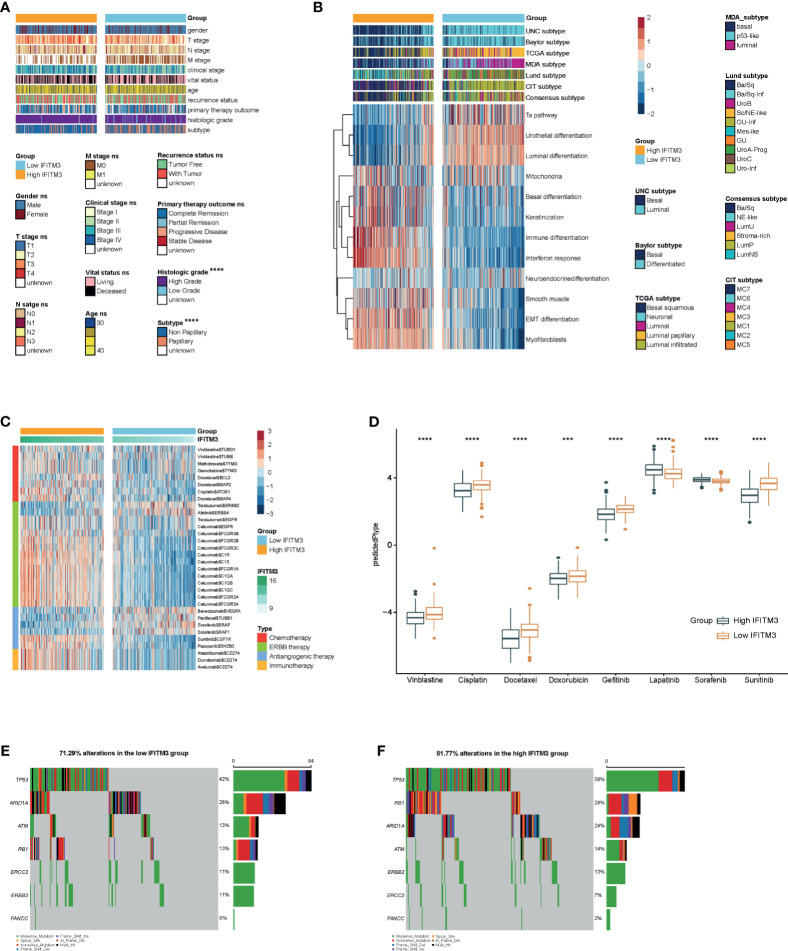
IFITM3 predicts the molecular subtype and response to therapeutic options in BLCA. **(A)** Correlations between IFITM3 and clinicopathological features in BLCA. **(B)** Correlations between IFITM3 and molecular subtypes using seven different algorithms (CIT, Lund, MDA, TCGA, Baylor, UNC, and consensus) and BLCA signatures. **(C)** Correlation between IFITM3 and the drug-target genes extracted from the DrugBank database. **(D)** Differences in the IC_50_ of common anticancer drugs calculated using the “pRRophetic” R package between the high- and low-IFITM3 groups. **(E, F)** Mutational profiles of chemotherapy-related genes in the high- and low-IFITM3 groups in the TCGA cohort. *p-value ≤ 0.05, **p-value ≤ 0.01, ***p-value ≤ 0.001, and ****p-value ≤ 0.0001.

Subsequently, we evaluated IFITM3 expression and the clinicopathological features of BLCA. As **Figure 4A** shows, IFITM3 was remarkably associated with histological grades (high grade vs. low grade) and subtypes (nonpapillary *vs* papillary) but not related to other features in the TCGA cohort (**Figure 4A**). Due to the lack of corresponding clinical information, these results could not be validated in the IMvigor210 cohort. Established molecular subtypes can predict the clinical response to immunotherapy, chemotherapy, and several targeted therapies (25). In the TCGA cohort, IFITM3 expression was associated with these subtypes, including the UNC subtype, consensus subtype, CIT subtype, Lund subtype, MDA subtype, and TCGA subtype (**Figure 4B**). Moreover, the enrichment scores for luminal differentiation, the Ta pathway, and urothelial differentiation were lower in the high-IFITM3 group. In addition, the enrichment scores for basal differentiation, EMT differentiation, immune infiltration, and interferon response were higher in the high-IFITM3 group (**Figure 4B**). These results were also partly confirmed in the IMvigor210 cohort (**Supplementary Figure S7A**). We also evaluated IFITM3 expression and the response to other therapies. The results from the DrugBank database (https://go.drugbank.com/) revealed a notably higher response to chemotherapy, anti-EGFR therapy, and immunotherapy in the high-IFITM3 group but a low response to anti-ERBB2, anti-ERBB4, and antiangiogenic therapy (**Figure 4C**, **Supplementary Figure S7B**). Moreover, the IC_50_ of anticancer drugs according to the pRRophetic algorithm was estimated. The results showed that patients with high IFITM3 expression were more sensitive to most anticancer drugs but resistant to lapatinib and sorafenib (not validated in the IMvigor210 cohort) (**Figure 4D**, **Supplementary Figure S7C**). In addition, the mutation rates of TP53, RB1, ERBB2, and FANCC were notably higher in the high-IFITM3 group (**Figures 4E, F**). Overall, IFITM3 is a novel classifier for the subtype of BLCA, and patients with high IFITM3 expression tend to be sensitive to more therapeutic options.

### IFITM3 Correlates With Emerging Immunobiomarkers in BLCA

RNA-seq data from the TCGA database could be applied to estimate microbial composition in tumor tissues (38). We comprehensively analyzed the correlations between microbiota abundance and the T-cell inflamed score. A total of three microbiota were extracted based on the criterion of the Pearson correlation coefficient ≥0.3 or ≤−0.3: Lachnoclostridium, Flammeovirga, and Terracter, which were positively correlated with the T-cell inflamed score (**Supplementary Figure S8A**). Next, the correlations between these immune-related microbiota abundances and the expression of immune checkpoints and immune cell abundance were assessed to validate their correlations with antitumor immunity. As expected, their abundance was positively correlated with the expression of immune checkpoints and immune cell abundance (**Supplementary Figures S8B, C**). These results implied that high abundances of Lachnoclostridium, Flammeovirga, and Terracter were correlated with a higher response to immunotherapy. Importantly, the abundances of Lachnoclostridium, Flammeovirga, and Terracter were notably higher in the high-IFITM3 group (**Supplementary Figure S8D**). Collectively, these results suggest that IFITM3 expression correlates with the response to immunotherapy from the view of correlations with immune-related microbiota.

**Figure 5:**
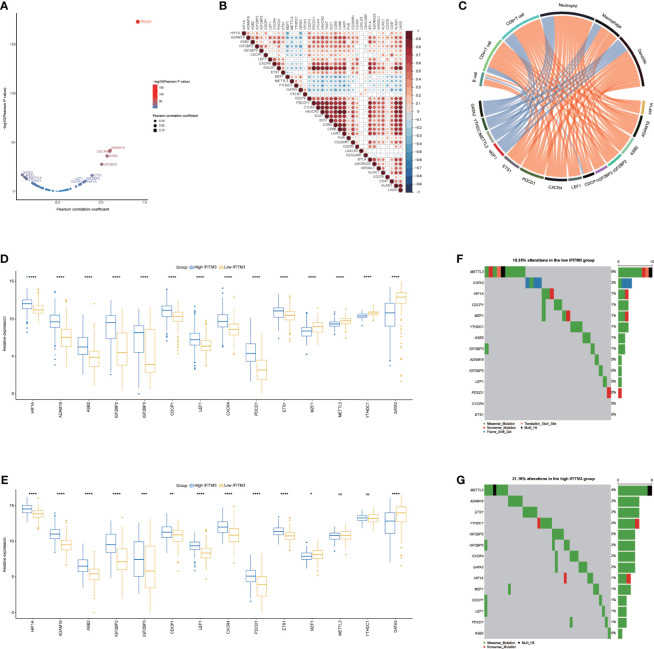
Correlation between IFITM3 and m6A genes in BLCA. **(A)** Correlation between the T cell inflamed score and m6A genes. The dots represent different m6A genes. The *y*-axis represents the Pearson correlation coefficient, while the *x*-axis represents −log_10_(*p*-value). **(B)** Correlations between immune-related m6A genes and immune checkpoint expression. **(C)** Correlations between immune-related m6A gene expression and TIIC levels estimated by TIMER. **(D, E)** Expression levels of immune-related m6A genes in the high- and low-IFITM3 groups. **(F, G)** Mutational profiles of immune-related m6A genes in the high- and low-IFITM3 groups in the TCGA cohort. *p-value ≤ 0.05, **p-value ≤ 0.01, ***p-value ≤ 0.001, and ****p-value ≤ 0.0001. NS: no statistical significance.

N6-methyladenosine (m6A), methylated at the N6 position of adenosine, plays crucial roles in the oncogenesis and progression of various cancers (39, 40). In addition, increased evidence suggests that m6A regulators are associated with antitumor immunity and could act as indicators for identifying tumor immunogenicity (41, 42). According to a recent publication (36), we extracted 64 m6A regulators and m6A interactive protein-coding genes with a high coexpression tendency. A total of 59 genes were expressed in BLCA, and among them, 46 genes were correlated with the T-cell inflamed score (**Supplementary Table S3**), indicating that m6A networks were tightly correlated with antitumor immunity. We extracted most related genes with the criterion of the Pearson correlation coefficient ≥0.3 or ≤−0.3 for further analysis (**Figure 5A**). These 10 m6A-related genes that positively correlated with the T-cell inflamed score were also positively correlated with immune checkpoint expression and immune cell infiltration, while the remaining four genes were negatively related to immune checkpoint expression and immune cell infiltration (**Figures 5B, C**). Next, the relationship between IFITM3 and these m6A genes was assessed. In the IFITM3 high group, 10 m6A-related genes that positively correlated with the T-cell inflamed score were significantly upregulated, and the remaining four genes were downregulated in the TCGA cohort (**Figure 5D**). In addition, most of these results were validated in the IMvigor210 cohort (**Figure 5E**). In addition, the mutation rates of immune-related m6A genes also varied in the high- and low-IFITM3 groups. In general, a high mutation rate was observed in the high-IFITM3 group (**Figures 5F, G**). In summary, m6A-related genes were remarkably related to antitumor immunity and were correlated with IFITM3.

### IFITM3 Is Associated With Immune and Clinical Phenotypes in the TMA Cohort

**Figure 6:**
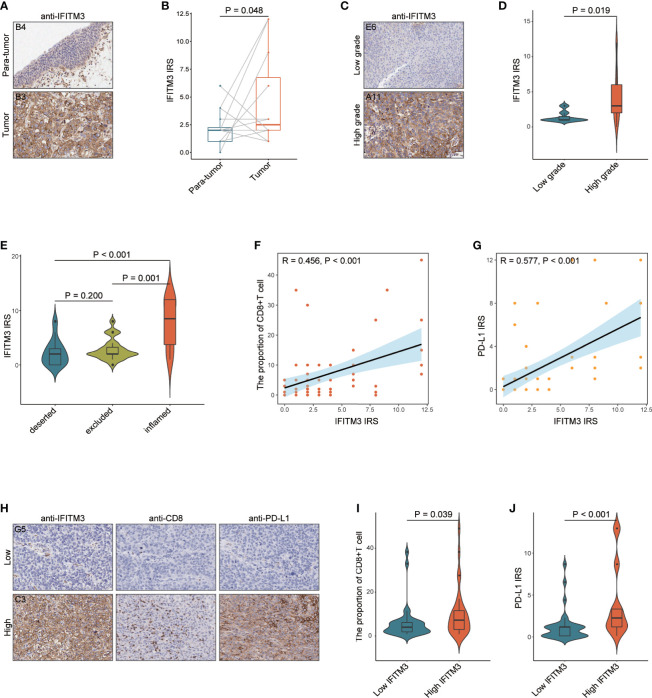
The role of IFITM3 in predicting clinical and immune phenotypes in the recruited TMA cohort. **(A)** Representative images revealing IFITM3 expression in tumor and paratumor tissues using anti-IFITM3 staining. Magnification, 200×. **(B)** Expression levels of IFITM3 in tumor and paratumor tissues. **(C)** Representative images revealing IFITM3 expression in high- and low-grade tumor tissues using anti-IFITM3 staining. Magnification, 200×. **(D)** Expression levels of IFITM3 in high- and low-grade tumor tissues. **(E)** Expression level of IFITM3 in the deserted, excluded, and inflamed phenotypes. **(F)** Correlation between IFITM3 expression and CD8+ T-cell infiltration levels. **(G)** Correlation between IFITM3 and PD-L1 expression. **(H)** Representative images revealing CD8+ T-cell infiltration and PD-L1 expression in the high- and low-IFITM3 groups. Magnification, 200×; **(I)** CD8+ T-cell infiltration level in the high- and low-IFITM3 groups. **(J)** Expression level of PD-L1 in the high- and low-IFITM3 groups.

To confirm the above results, we also obtained a TMA cohort for validation, which included 63 BLCA and 16 paracancerous samples (**Supplementary Figures S9A–D**). There was no significant difference between BLCA and paracancerous tissues in terms of IFITM3 expression (**Figures 6A, B**). However, IFITM3 was more highly expressed in high-grade BLCA than in low-grade BLCA (**Figure 6C, D**). Next, BLCA samples were divided into the inflamed phenotype, the excluded phenotype, and the deserted phenotype according to the spatial distribution of CD8+ T cells (**Supplementary Figure S10A**), and we found that CD8+ T-cell infiltration and PD-L1 expression notably varied in these subtypes (**Supplementary Figures S10B, C**). We also found that IFITM3 exhibited the highest expression in the inflamed phenotype and the lowest expression in the deserted phenotype, which was similar to the expression pattern of PD-L1 (**Figure 6E**). Moreover, IFITM3 was positively correlated with CD8+ T-cell infiltration and PD-L1 expression in the current cohort (**Figures 6F, G**). In addition, the current BLCA cohort was classified into low- and high-expression groups based on the median level of IFITM3 expression (IRS ≤ 2 vs. IRS > 3), and we found that the infiltrating level of CD8+ T cells and PD-L1 expression were higher in the high-IFITM3 group (**Figures 6H–J**). Overall, IFITM3 expression is correlated with immune phenotypes and clinical features in BLCA.

### Extension of Immunological Role of IFITM3 in Pancancer

All these data illustrated that IFITM3 produced an inflamed TME and could be used to identify immuno-hot tumors in BLCA. However, as previously shown in **Figure 1**, except for several tumor types, IFITM3 may be a pancancer indicator for tumor immunogenicity. We analyzed the correlation between IFITM3 and chemokine, MHC, immunostimulator, and receptor scores. Except for THYM and MESO, IFITM3 was positively correlated with the chemokine score (**Supplementary Figure S11A**); except for TGCT, MESO, CHOL, and READ, IFITM3 was positively correlated with the MHC score (**Supplementary Figure S11B**); except for TGCT, THYM, MESO, CHOL, READ, and DLBC, IFITM3 was positively correlated with the immunostimulator score (**Supplementary Figure S11C**); except for TGCT, THYM, MESO, CHOL, LAML, READ, and DLBC, IFITM3 was positively correlated with MHC score (**Supplementary Figure S11D**). In addition, we also assessed the correlation between IFITM3 and the T-cell inflamed score. The results showed that IFITM3 was positively correlated with the T-cell inflamed score in most cancers except for TGCT, MESO, CHOL, and READ (**Supplementary Figure S11E**). Taken together, these data suggest that IFITM3 is a pancancer classifier for high immunogenicity except for a few tumor types.

## Discussion

According to previous studies, IFITM3 is an oncogene and promotes tumor progression in the majority of cancers but also exerts a tumor suppressive role in several cancers (6). Early research revealed that IFITM3 is a tumor biomarker, followed by its observed upregulation in colon cancer (43). In the following years, upregulation of IFITM3 in tumor tissues in comparison with paratumor tissues was confirmed in multiple cancers, such as gastric cancer (44), breast cancer (45), prostate cancer (46), and lung cancer (47). In addition, upregulation of IFITM3 was also found in precancerous tissues, such as in ulcerative colitis (48). IFITM3 is expected to be overexpressed under inflammatory conditions, given that interferon is one of most important regulators of inflammatory processes. However, IFITM3 is also overexpressed in acute myeloid leukemia and predicts adverse prognosis (49), supporting the assumption that IFITM3 overexpression is a general hallmark of human cancers and not just inflammation. Theoretically, considering that IFITM3 is a crucial responder to inflammation to some extent, IFITM3 may play a significant role in regulating tumor immunity, but its role as an immunomodulator in human cancers has not been well defined.

In this study, we performed a pancancer analysis of IFITM3 by using large-scale RNA-seq data and performed an experiment to validate the role of IFITM3 in BLCA. We found that most types of cancer expressed higher IFITM3 than normal tissues, which was in accordance with previous reports. The expression of IFITM3 in BLCA found in public databases was inconsistent, but IFITM3 was highly expressed in the tumor tissues compared with paired paratumor tissues in the current cohort. Regarding the prognostic value of IFITM3, high expression of IFITM3 was associated with better prognosis in KIRC and glioma, while in SKCM, IFITM3 was a risk prognostic factor. However, the functional role of IFITM3 in BLCA needs to be further investigated.

More importantly, IFITM3 expression was positively related to immunomodulator (including chemokine, MHC, immunostimulator, and receptor) and TIIC levels across cancers. Furthermore, IFITM3 exhibited the tightest correlation with immunofactors in BLCA. Specifically, we discovered that IFITM3 was positively correlated with the expression of critical immunomodulators, such as CCL5, CXCL9, and CXCL10, as well as the activities of the cancer-immunity cycle. To some extent, the immunological role of IFITM3 was opposite to several reported immunosuppressive oncogenic pathways, such as the β-catenin, PPAR-γ, and FGFR3 pathways (50–52). These pathways have been revealed to suppress the infiltration of TIICs by decreasing the expression of immunomodulators, contributing to a noninflamed TME. IFITM3 was remarkably negatively correlated with the enrichment scores of these oncogenic pathways but positively correlated with immunopermissive pathways, such as the IFN-γ signature, EGFR ligands, and hypoxia. However, immune cells, such as B cells, also express IFITM3 (53). Thus, bulk RNA-seq data were obtained from immune cells and nonimmune cells, resulting in inaccurate results of its correlation with immune infiltration. In our validated cohort, we only assessed the expression of IFITM3 in cancer cells, and IFITM3 expression exhibited tight correlations with PD-L1 expression in tumor cells and the infiltration of CD8+ T cells. In addition, IFITM3 was correlated with emerging immunobiomarkers in BLCA, including immune-related microbiota and immune-related m6A regulators and m6A interactive protein-coding genes. However, it remains to be further clarified whether IFITM3 is merely a biomarker for tumor immunogenicity or has a regulatory effect on antitumor immunity.

Molecular subtype can explain the heterogeneity of BLCA at the molecular level; thus, molecular subtype can be used as a classifier to predict the clinical outcome and the response to therapeutic opportunities (54). Encouragingly, IFITM3 predicted multiple molecular subtypes in BLCA, including the UNC subtype, consensus subtype, CIT subtype, lung subtype, MDA subtype, and TCGA subtype. In addition, the enrichment scores for luminal differentiation, the Ta pathway, and urothelial differentiation were lower, and the enrichment scores for immune differentiation, interferon response, and EMT differentiation were higher in the high-IFITM3 group. Moreover, IFITM3 predicted the response to therapeutic options in BLCA, and patients with high IFITM3 expression exhibited a high response to chemotherapy, anti-EGFR therapy, and immunotherapy but a low response to anti-ERBB2, anti-ERBB4, and antiangiogenic therapy. In general, high expression of IFITM3 produced an inflamed TME, identified immuno-hot tumors, and predicted a better response to more therapeutic options. Theoretically, high IFITM3 expression should predict better prognosis. However, IFITM3 also upregulated several oncogenic molecules, including C-MYC, CCND1, CDK4, and MMP9 (7, 55, 56). Thus, IFITM3 may function as a friend or foe in cancer, and understanding this balancing act may be an interesting hotspot for further research.

## Conclusions

The current study reveals that IFITM3 expression shapes an inflamed TME in BLCA and can predict the immune and clinical phenotypes in BLCA. Moreover, the pancancer analysis suggests that IFITM3 is an identifying factor for high immunogenicity in most cancers. Overall, IFITM3 might be a promising biomarker for identifying tumor immunogenicity and guiding immunotherapy.

## Data Availability Statement

The original contributions presented in the study are included in the article/**Supplementary Material**. Further inquiries can be directed to the corresponding authors.

## Ethics Statement

The studies involving human participants were reviewed and approved by the Clinical Research Ethics Committee, Outdo Biotech (Shanghai, China).

## Author Contributions

JM, HZ, and JY conceived the study and participated in the study design, performance, coordination, and project supervision. YC, WJ, CS, XC, YD, and JP collected the public data and performed the bioinformatics analysis. JM and YC performed the IHC staining. JM, HZ, and JY revised the manuscript. All authors contributed to the article and approved the submitted version.

## Funding

This work was supported by the General Project of Wuxi Health Commission (M202016) and the Jiangsu Postgraduate Training Innovation Project (KYCX21-1559).

## Conflict of Interest

The authors declare that the research was conducted in the absence of any commercial or financial relationships that could be construed as a potential conflict of interest.

## Publisher’s Note

All claims expressed in this article are solely those of the authors and do not necessarily represent those of their affiliated organizations, or those of the publisher, the editors and the reviewers. Any product that may be evaluated in this article, or claim that may be made by its manufacturer, is not guaranteed or endorsed by the publisher.

## Abbreviations

COVID-19, Coronavirus Disease 2019; SARS-CoV-2, Severe Acute Respiratory Syndrome Coronavirus 2; ACE2, angiotensin-converting enzyme 2; IFITM3, interferon-inducible transmembrane protein 3; TMPRSS2, transmembrane serine protease 2; EMT, epithelial-to-mesenchymal transition; TME, tumor microenvironment; BLCA, bladder cancer; TCGA, The Cancer Genome Atlas; CNV, copy number variant; GO, Gene Ontology; KEGG, Kyoto Encyclopedia of Genes and Genomes; GSEA, gene set enrichment analysis; TIICs, tumor-infiltrating immune cells; ssGSEA, single sample gene set enrichment analysis; CGP, Cancer Genome Project; IC_50_, half-maximal inhibitory concentration; TMA, tissue microarray; IHC, immunohistochemistry; IRS, immunoreactivity score; OS, overall survival; PFS, progression-free survival; IC, immune cells; TC, tumor cells; m6A, N6-methyladenosine.
