# Supplementary material for: Interferon-Induced Transmembrane Protein 3 Shapes an Inflamed Tumor Microenvironment and Identifies Immuno-Hot Tumors
Source: Front Immunol. 2021 Aug 11;12:704965. doi: 10.3389/fimmu.2021.704965 (PMC8385493; doi:10.3389/fimmu.2021.704965)
Supplement: Supplementary file 1 [file DataSheet_1.pdf]

Supplementary Figures and Tables

Figure S1. Pan-cancer analysis of expression and prognostic value of IFITM3.

(A) Expression of IFITM3 across different cancer types. The thresholds of differential expression were set as follows: analysis type, cancer vs. normal; threshold (p value), 0.05; threshold (fold-change), all; threshold (gene rank), top 10%; and data type, mRNA. (B) Pan-cancer analysis of IFITM3 expression in tumor and normal tissues in the TCGA database. Forest plots of prognostic value of IFITM3 in predicting (C) OS and (D) PFS in pan-cancer analysis.

Figure S2. Correlation between IFITM3 and common immune checkpoints.

(A) PDCD1, (B) HAVCR, (C) CD80, (D) CD86. The dots represent cancer types. The Y-axis represents the Pearson correlation coefficient, while the X-axis represents $-\log_{10}$ (P value).

Figure S3. Potential regulatory factors of IFITM3 in BLCA.

(A) Exhibition of mutations in IFITM3 gene. (B) The associations between CNV pattern and IFITM3 expression in BLCA. (C) The correlation between methylation levels of single sites and IFITM3 expression.

Figure S4. IFITM3 shapes an inflamed TME in BLCA (IMvigor210 cohort).

(A, B) Expression levels of 122 immunomodulators (chemokines, immunostimulators, MHC and receptors) in the high and low IFITM3 groups in BLCA. (C) Distribution of Tumor Purity, ESTIMATE Score, Immune Score and Stromal Score calculating using ESTIMATE algorithm in the high and low IFITM3 groups. (D) The levels of TIICs calculated using five algorithms (TIMER, EPIC, MCP-counter, quanTIseq and TISIDB) in the high and low IFITM3 groups. (E) Expression levels of the gene markers of the common TIICs in the high and low IFITM3 groups. (F) The activities of the various steps of the cancer immunity cycle calculated by ssGSEA algorithm in the high and low IFITM3 groups. (G) Correlations between IFITM3 and common inhibitory immune checkpoints. The color and the values indicate the Pearson

correlation coefficient.

Figure S5. Hypoxia scores in the high and low IFITM3 groups.

Difference in (A) Buffa, (B) Winter and (C) Ragnum hypoxia scores in the high and low IFITM3 groups. These scores were obtained from the ciboPortal website.

Figure S6. Predictive values of several indicators for response to immunotherapy in the IMvigor210 cohort.

(A) IFITM3, (B) CD274, (C) CTLA4, (D) PDCD1, (E) PVR, (F) TIGIT.

Figure S7. IFITM3 predicts the molecular subtype and the therapeutic options in BLCA (IMvigor210 cohort).

(A) Correlations between IFITM3 and molecular subtypes BLCA signatures. (B) Correlation between IFITM3 and the drug-target genes extracted from the Drugbank database. (C) Differences in IC50 of common anti-cancer drugs calculated using the R package "pRRophetic" between the high and low IFITM3 groups.

Figure S8. Correlation between IFITM3 and microbiome signature in BLCA.

(A) Correlation between T cell inflamed score and tumor microbial abundances. The dots represent different microbiota. The Y-axis represents the Pearson correlation coefficient, while the X-axis represents $-\log_{10}$(P value). (B) Correlations between immune-related microbiota abundances and immune checkpoints expression. (C) Correlations between immune-related microbiota abundances and TIICs levels estimated by TIMER. (D) Differences in the abundances of immune-related microbiota between the high and low IFITM3 groups.

Figure S9. The landscape of TMA HBlaU079Su01.

(A) Distribution of samples in TMA HBlaU079Su01. Blue dots: tumor samples; Yellow dots: para-tumor samples. (B) The landscape of anti-IFITM3 staining TMA HBlaU079Su01. (C) The landscape of anti-PD-L1 staining TMA HBlaU079Su01. (D)

The landscape of anti-CD8 staining TMA HBlaU079Su01.

Figure S10. Subgrouping of three immune subtypes in BLCA.

(A) Representative microphotographs revealing the deserted, the excluded and the inflamed phenotypes using anti-CD8 staining. Magnification, 200X. (B) Infiltrating level of CD8+T cells in these sub-phenotypes. (C) Expression level of PD-L1 expressed on tumor cells in these sub-phenotypes.

Figure S11. Pan-caner analysis of immunological role of IFITM3

(A) Pan-cancer correlations between IFITM3 and chemokine score. (B) Pan-cancer correlations between IFITM3 and MHC score. (C) Pan-cancer correlations between IFITM3 and immunostimulator score. (D) Pan-cancer correlations between IFITM3 and receptor score. (E) Pan-cancer correlations between IFITM3 and T cell inflamed score.

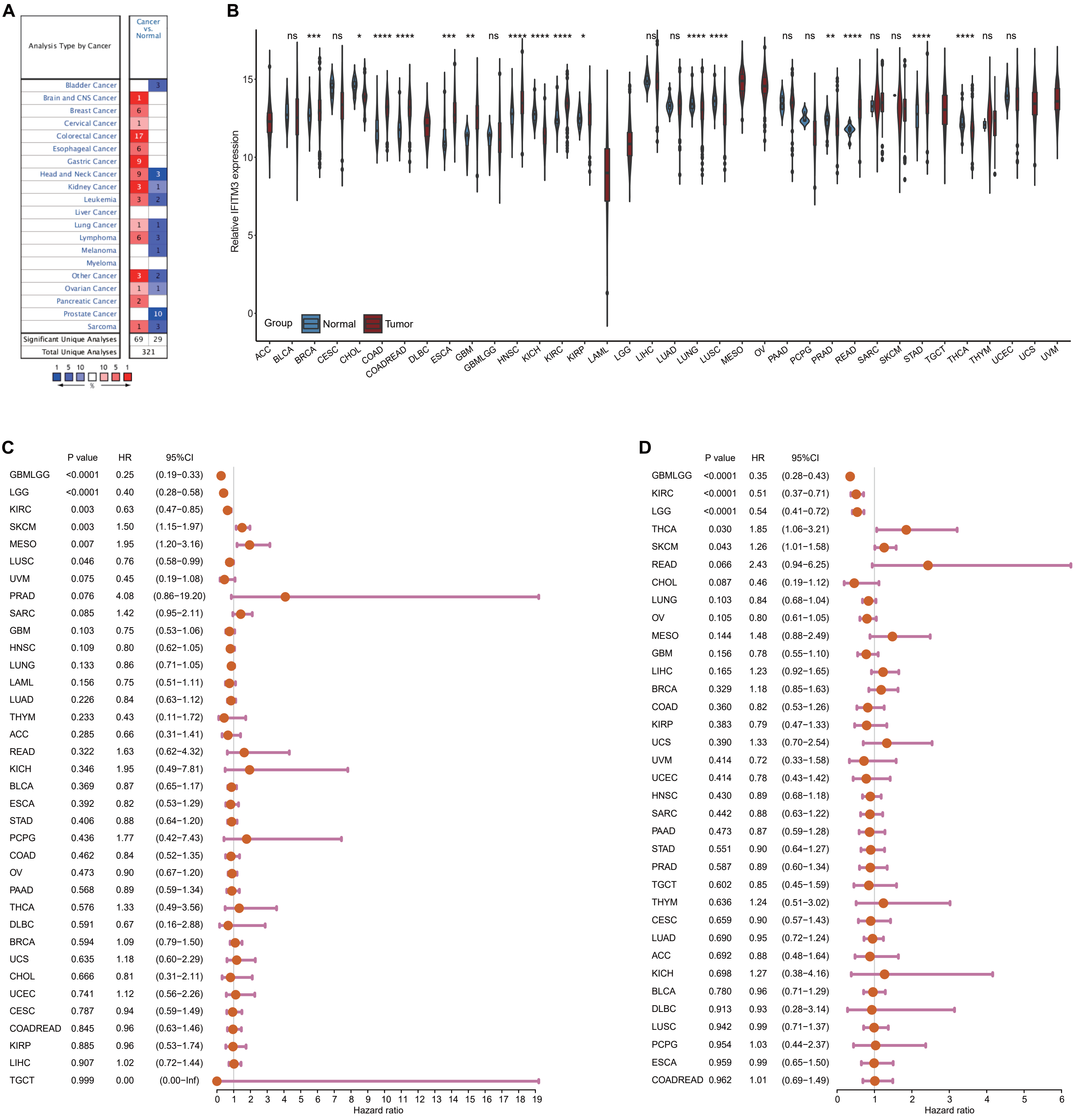
A
Analysis Type by Cancer
Cancer vs. Normal
Bladder Cancer 3
Brain and CNS Cancer 1
Breast Cancer 6
Cervical Cancer 1
Colorectal Cancer 17
Esophageal Cancer 6
Gastric Cancer 9
Head and Neck Cancer 9 3
Kidney Cancer 3 1
Leukemia 3 2
Liver Cancer
Lung Cancer 1 1
Lymphoma 6 3
Melanoma 1
Myeloma
Other Cancer 3 2
Ovarian Cancer 1 1
Pancreatic Cancer 2
Prostate Cancer 10
Sarcoma 1 3
Significant Unique Analyses 69 29
Total Unique Analyses 321
1 5 10 10 5 1
%
B
Relative IFITM3 expression
Group Normal Tumor
ns *** ns * **** **** *** ** ns **** **** **** * ns ns **** **** ns ns ** **** ns ns **** **** ns ns
ACC BLCA BRCA CESC CHOL COAD COADREAD DLBC ESCA GBM GBMLGG HNSC KICH KIRC KIRP LAML LGG LIHC LUAD LUNG LUSC MESO OV PAAD PCPG PRAD READ SARC SKCM STAD TGCT THCA THYM UCEC UCS UVM
C
P value HR 95%CI
GBMLGG <0.0001 0.25 (0.19−0.33)
LGG <0.0001 0.40 (0.28−0.58)
KIRC 0.003 0.63 (0.47−0.85)
SKCM 0.003 1.50 (1.15−1.97)
MESO 0.007 1.95 (1.20−3.16)
LUSC 0.046 0.76 (0.58−0.99)
UVM 0.075 0.45 (0.19−1.08)
PRAD 0.076 4.08 (0.86−19.20)
SARC 0.085 1.42 (0.95−2.11)
GBM 0.103 0.75 (0.53−1.06)
HNSC 0.109 0.80 (0.62−1.05)
LUNG 0.133 0.86 (0.71−1.05)
LAML 0.156 0.75 (0.51−1.11)
LUAD 0.226 0.84 (0.63−1.12)
THYM 0.233 0.43 (0.11−1.72)
ACC 0.285 0.66 (0.31−1.41)
READ 0.322 1.63 (0.62−4.32)
KICH 0.346 1.95 (0.49−7.81)
BLCA 0.369 0.87 (0.65−1.17)
ESCA 0.392 0.82 (0.53−1.29)
STAD 0.406 0.88 (0.64−1.20)
PCPG 0.436 1.77 (0.42−7.43)
COAD 0.462 0.84 (0.52−1.35)
OV 0.473 0.90 (0.67−1.20)
PAAD 0.568 0.89 (0.59−1.34)
THCA 0.576 1.33 (0.49−3.56)
DLBC 0.591 0.67 (0.16−2.88)
BRCA 0.594 1.09 (0.79−1.50)
UCS 0.635 1.18 (0.60−2.29)
CHOL 0.666 0.81 (0.31−2.11)
UCEC 0.741 1.12 (0.56−2.26)
CESC 0.787 0.94 (0.59−1.49)
COADREAD 0.845 0.96 (0.63−1.46)
KIRP 0.885 0.96 (0.53−1.74)
LIHC 0.907 1.02 (0.72−1.44)
TGCT 0.999 0.00 (0.00−Inf)
Hazard ratio
D
P value HR 95%CI
GBMLGG <0.0001 0.35 (0.28−0.43)
KIRC <0.0001 0.51 (0.37−0.71)
LGG <0.0001 0.54 (0.41−0.72)
THCA 0.030 1.85 (1.06−3.21)
SKCM 0.043 1.26 (1.01−1.58)
READ 0.066 2.43 (0.94−6.25)
CHOL 0.087 0.46 (0.19−1.12)
LUNG 0.103 0.84 (0.68−1.04)
OV 0.105 0.80 (0.61−1.05)
MESO 0.144 1.48 (0.88−2.49)
GBM 0.156 0.78 (0.55−1.10)
LIHC 0.165 1.23 (0.92−1.65)
BRCA 0.329 1.18 (0.85−1.63)
COAD 0.360 0.82 (0.53−1.26)
KIRP 0.383 0.79 (0.47−1.33)
UCS 0.390 1.33 (0.70−2.54)
UVM 0.414 0.72 (0.33−1.58)
UCEC 0.414 0.78 (0.43−1.42)
HNSC 0.430 0.89 (0.68−1.18)
SARC 0.442 0.88 (0.63−1.22)
PAAD 0.473 0.87 (0.59−1.28)
STAD 0.551 0.90 (0.64−1.27)
PRAD 0.587 0.89 (0.60−1.34)
TGCT 0.602 0.85 (0.45−1.59)
THYM 0.636 1.24 (0.51−3.02)
CESC 0.659 0.90 (0.57−1.43)
LUAD 0.690 0.95 (0.72−1.24)
ACC 0.692 0.88 (0.48−1.64)
KICH 0.698 1.27 (0.38−4.16)
BLCA 0.780 0.96 (0.71−1.29)
DLBC 0.913 0.93 (0.28−3.14)
LUSC 0.942 0.99 (0.71−1.37)
PCPG 0.954 1.03 (0.44−2.37)
ESCA 0.959 0.99 (0.65−1.50)
COADREAD 0.962 1.01 (0.69−1.49)
Hazard ratio

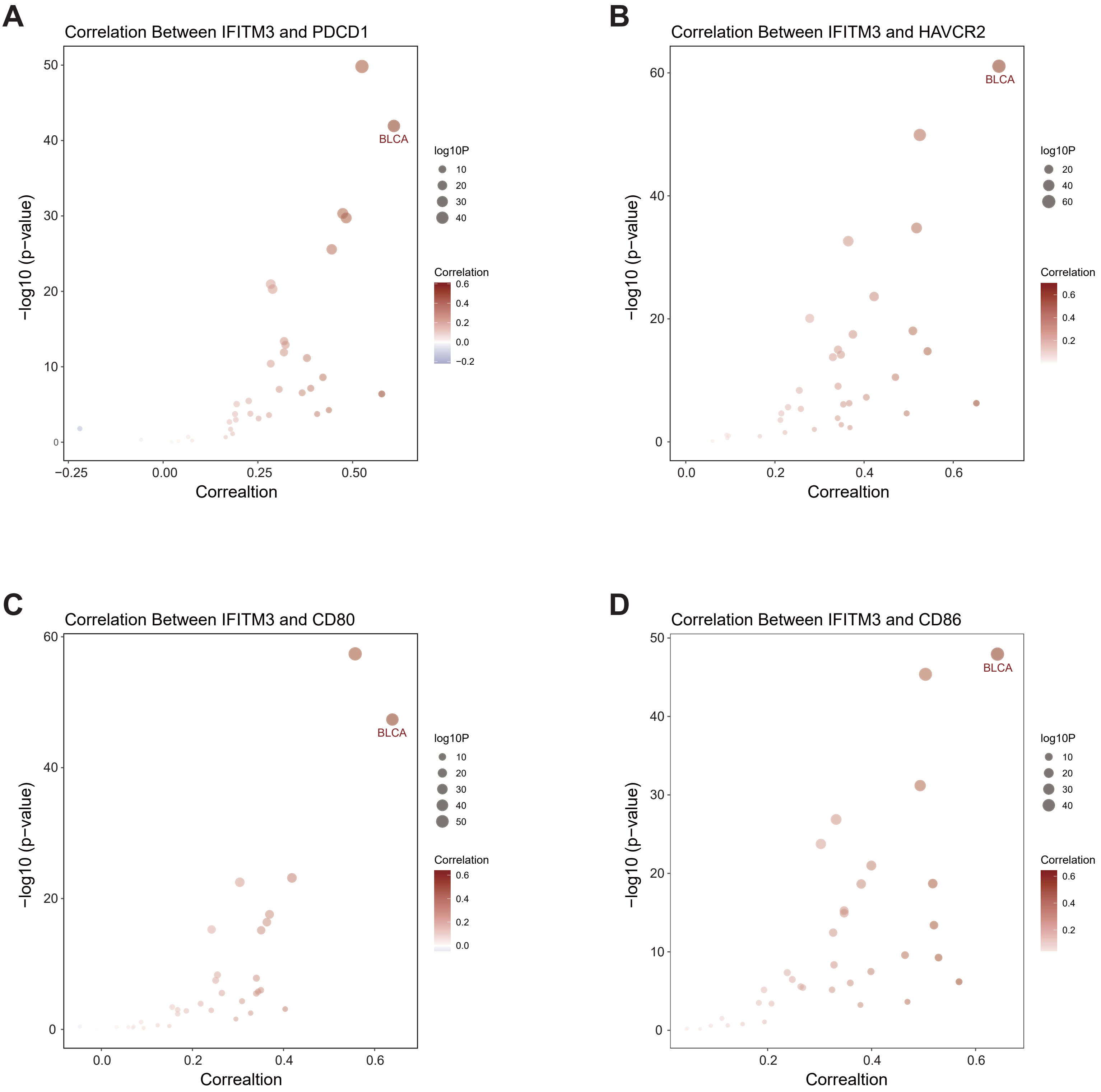
A
Correlation Between IFITM3 and PDCD1
−log10 (p−value)
Correaltion
BLCA
log10P
10
20
30
40
Correlation
0.6
0.4
0.2
0.0
−0.2
B
Correlation Between IFITM3 and HAVCR2
−log10 (p−value)
Correaltion
BLCA
log10P
20
40
60
Correlation
0.6
0.4
0.2
C
Correlation Between IFITM3 and CD80
−log10 (p−value)
Correaltion
BLCA
log10P
10
20
30
40
50
Correlation
0.6
0.4
0.2
0.0
D
Correlation Between IFITM3 and CD86
−log10 (p−value)
Correaltion
BLCA
log10P
10
20
30
40
Correlation
0.6
0.4
0.2

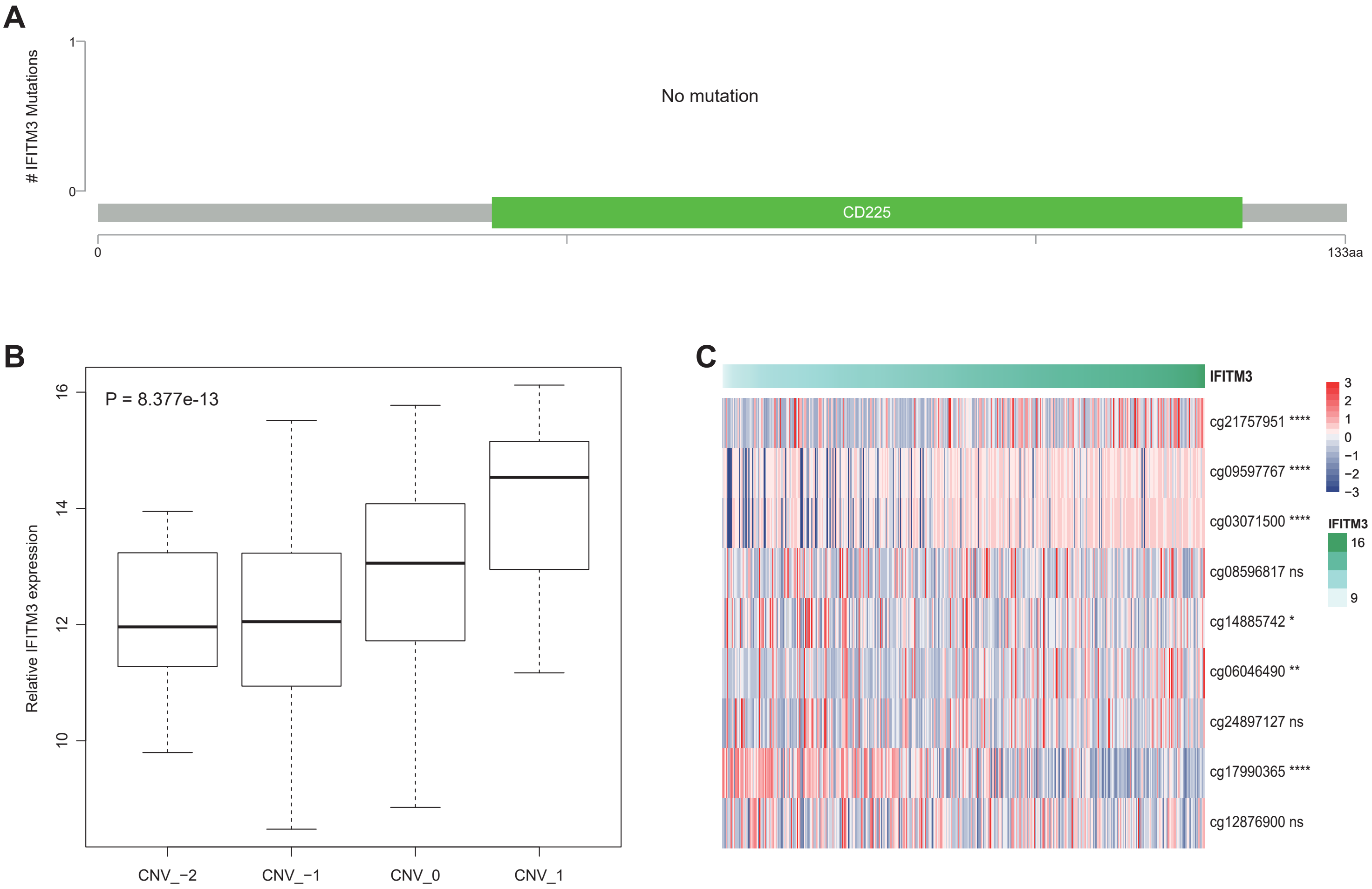
A
IFITM3 Mutations
1
0
No mutation
CD225
0
133aa
B
P = 8.377e-13
Relative IFITM3 expression
16
14
12
10
CNV_−2
CNV_−1
CNV_0
CNV_1
C
IFITM3
cg21757951 ****
cg09597767 ****
cg03071500 ****
cg08596817 ns
cg14885742 *
cg06046490 **
cg24897127 ns
cg17990365 ****
cg12876900 ns
3
2
1
0
−1
−2
−3
IFITM3
16
9

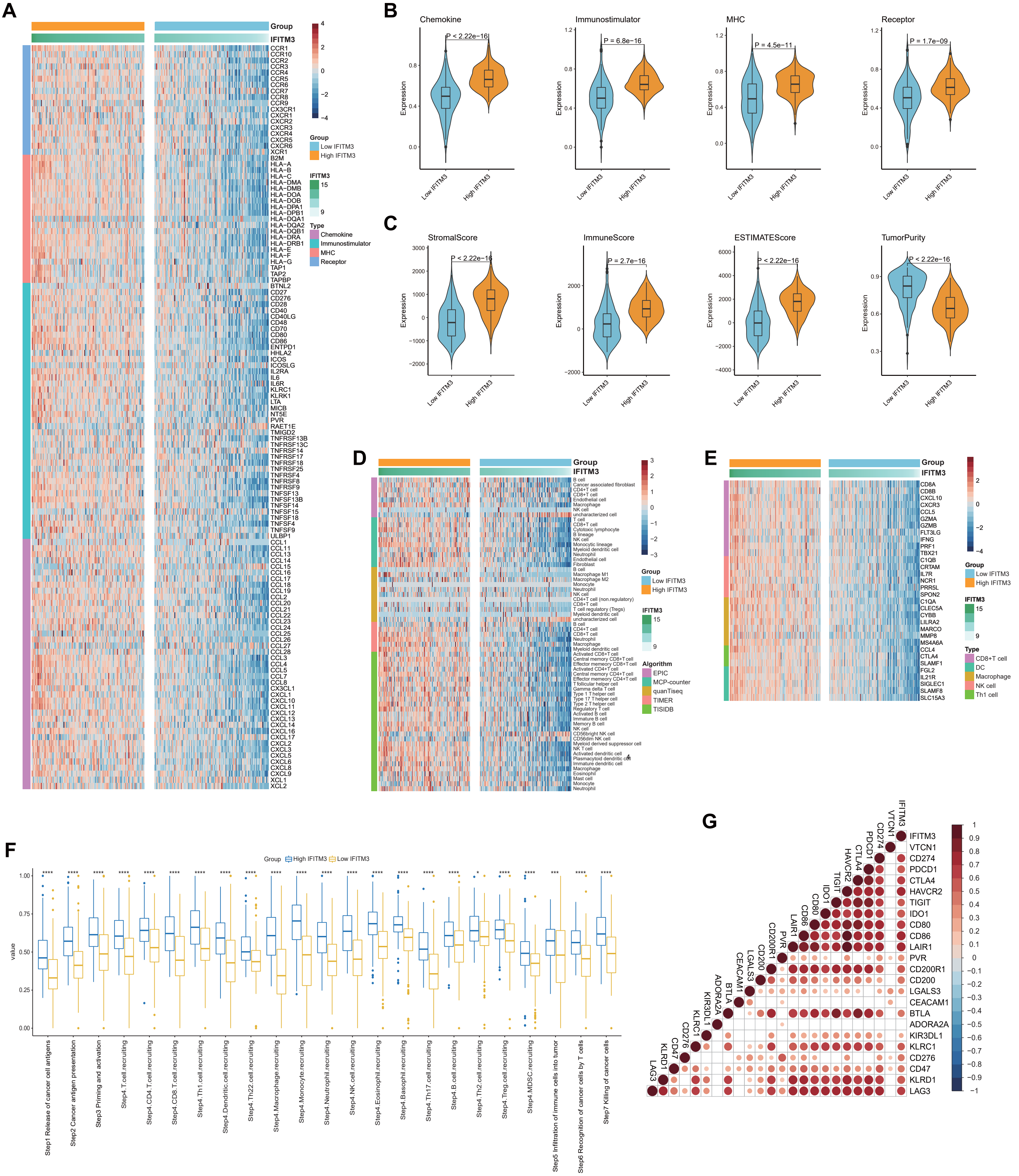
A
Group
IFITM3
CCR1
CCR10
CCR2
CCR3
CCR4
CCR5
CCR6
CCR7
CCR8
CCR9
CX3CR1
CXCR1
CXCR2
CXCR3
CXCR4
CXCR5
CXCR6
XCR1
B2M
HLA−A
HLA−B
HLA−C
HLA−DMA
HLA−DMB
HLA−DOA
HLA−DOB
HLA−DPA1
HLA−DPB1
HLA−DQA1
HLA−DQA2
HLA−DQB1
HLA−DRA
HLA−DRB1
HLA−E
HLA−F
HLA−G
TAP1
TAP2
TAPBP
BTNL2
CD27
CD276
CD28
CD40
CD40LG
CD48
CD70
CD80
CD86
ENTPD1
HHLA2
ICOS
ICOSLG
IL2RA
IL6
IL6R
KLRC1
KLRK1
LTA
MICB
NT5E
PVR
RAET1E
TMIGD2
TNFRSF13B
TNFRSF13C
TNFRSF14
TNFRSF17
TNFRSF18
TNFRSF25
TNFRSF4
TNFRSF8
TNFRSF9
TNFSF13
TNFSF13B
TNFSF14
TNFSF15
TNFSF18
TNFSF4
TNFSF9
ULBP1
CCL1
CCL11
CCL13
CCL14
CCL15
CCL16
CCL17
CCL18
CCL19
CCL2
CCL20
CCL21
CCL22
CCL23
CCL24
CCL25
CCL26
CCL27
CCL28
CCL3
CCL4
CCL5
CCL7
CCL8
CX3CL1
CXCL1
CXCL10
CXCL11
CXCL12
CXCL13
CXCL14
CXCL16
CXCL17
CXCL2
CXCL3
CXCL5
CXCL6
CXCL8
CXCL9
XCL1
XCL2
4
2
0
−2
−4
Group
Low IFITM3
High IFITM3
IFITM3
15
9
Type
Chemokine
Immunostimulator
MHC
Receptor
B
Chemokine
P < 2.22e−16
Immunostimulator
P = 6.8e−16
MHC
P = 4.5e−11
Receptor
P = 1.7e−09
Expression
Low IFITM3
High IFITM3
C
StromalScore
P < 2.22e−16
ImmuneScore
P = 2.7e−16
ESTIMATEScore
P < 2.22e−16
TumorPurity
P < 2.22e−16
Expression
Low IFITM3
High IFITM3
D
Group
IFITM3
B cell
Cancer associated fibroblast
CD4+T cell
CD8+T cell
Endothelial cell
Macrophage
NK cell
uncharacterized cell
T cell
CD8+T cell
Cytotoxic lymphocyte
B lineage
NK cell
Monocytic lineage
Myeloid dendritic cell
Neutrophil
Endothelial cell
Fibroblast
B cell
Macrophage M1
Macrophage M2
Monocyte
Neutrophil
NK cell
CD4+T cell (non.regulatory)
CD8+T cell
T cell regulatory (Tregs)
Myeloid dendritic cell
uncharacterized cell
B cell
CD4+T cell
CD8+T cell
Neutrophil
Macrophage
Myeloid dendritic cell
Activated CD8+T cell
Central memory CD8+T cell
Effector memeory CD8+T cell
Activated CD4+T cell
Central memory CD4+T cell
Effector memeory CD4+T cell
T follicular helper cell
Gamma delta T cell
Type 1 T helper cell
Type 17 T helper cell
Type 2 T helper cell
Regulatory T cell
Activated B cell
Immature B cell
Memory B cell
NK cell
CD56bright NK cell
CD56dim NK cell
Myeloid derived suppressor cell
NK T cell
Activated dendritic cell
Plasmacytoid dendritic cell
Immature dendritic cell
Macrophage
Eosinophil
Mast cell
Monocyte
Neutrophil
3
2
1
0
−1
−2
−3
Group
Low IFITM3
High IFITM3
IFITM3
15
9
Algorithm
EPIC
MCP-counter
quanTIseq
TIMER
TISIDB
E
Group
IFITM3
CD8A
CD8B
CXCL10
CXCR3
CCL5
GZMA
GZMB
FLT3LG
IFNG
PRF1
TBX21
C1QB
CRTAM
IL7R
NCR1
PRR5L
SPON2
C1QA
CLEC5A
CYBB
LILRA2
MARCO
MMP8
MS4A6A
CCL4
CTLA4
SLAMF1
FGL2
IL21R
SIGLEC1
SLAMF8
SLC15A3
2
0
−2
−4
Group
Low IFITM3
High IFITM3
IFITM3
15
9
Type
CD8+T cell
DC
Macrophage
NK cell
Th1 cell
F
Group
High IFITM3
Low IFITM3
value
Step1 Release of cancer cell antigens
Step2 Cancer antigen presentation
Step3 Priming and activation
Step4.T.cell.recruiting
Step4.CD4.T.cell.recruiting
Step4.CD8.T.cell.recruiting
Step4.Th1.cell.recruiting
Step4.Dendritic.cell.recruiting
Step4.Th22.cell.recruiting
Step4.Macrophage.recruiting
Step4.Monocyte.recruiting
Step4.Neutrophil.recruiting
Step4.NK.cell.recruiting
Step4.Eosinophil.recruiting
Step4.Basophil.recruiting
Step4.Th17.cell.recruiting
Step4.B.cell.recruiting
Step4.Th2.cell.recruiting
Step4.Treg.cell.recruiting
Step4.MDSC.recruiting
Step5 Infiltration of immune cells into tumor
Step6 Recognition of cancer cells by T cells
Step7 Killing of cancer cells
G
IFITM3
VTCN1
CD274
PDCD1
CTLA4
HAVCR2
TIGIT
IDO1
CD80
CD86
LAIR1
PVR
CD200R1
CD200
LGALS3
CEACAM1
BTLA
ADORA2A
KIR3DL1
KLRC1
CD276
CD47
KLRD1
LAG3
1
0.9
0.8
0.7
0.6
0.5
0.4
0.3
0.2
0.1
0
−0.1
−0.2
−0.3
−0.4
−0.5
−0.6
−0.7
−0.8
−0.9
−1

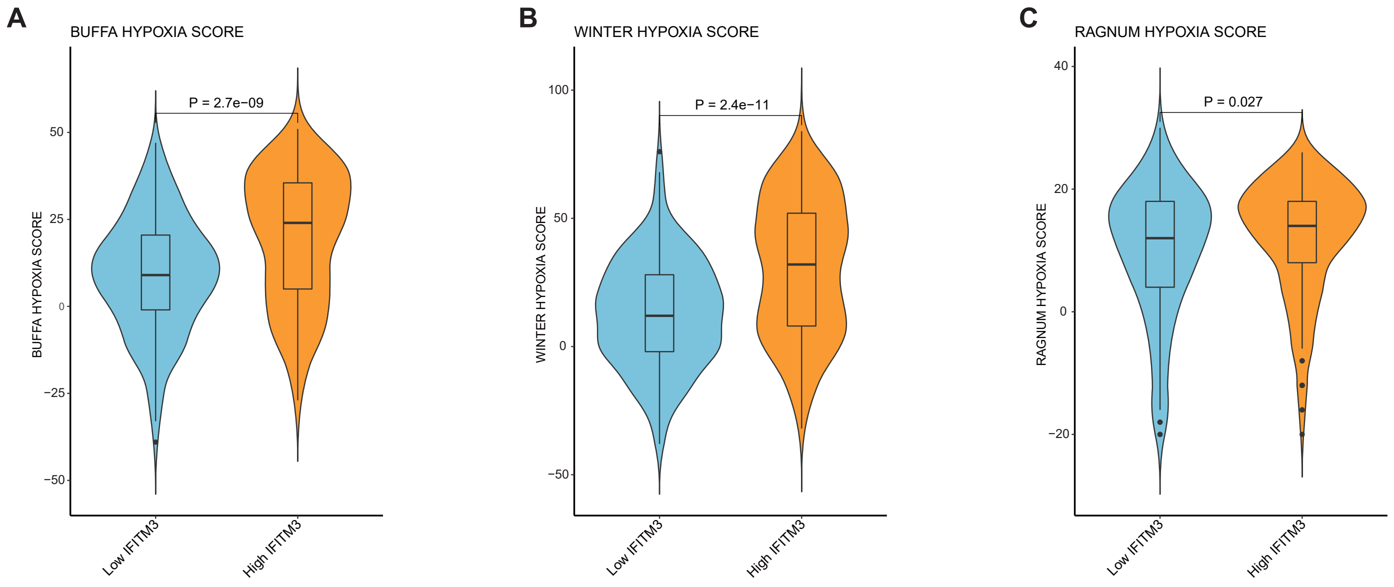
A
BUFFA HYPOXIA SCORE
P = 2.7e−09
BUFFA HYPOXIA SCORE
50
25
0
−25
−50
Low IFITM3
High IFITM3
B
WINTER HYPOXIA SCORE
P = 2.4e−11
WINTER HYPOXIA SCORE
100
50
0
−50
Low IFITM3
High IFITM3
C
RAGNUM HYPOXIA SCORE
P = 0.027
RAGNUM HYPOXIA SCORE
40
20
0
−20
Low IFITM3
High IFITM3

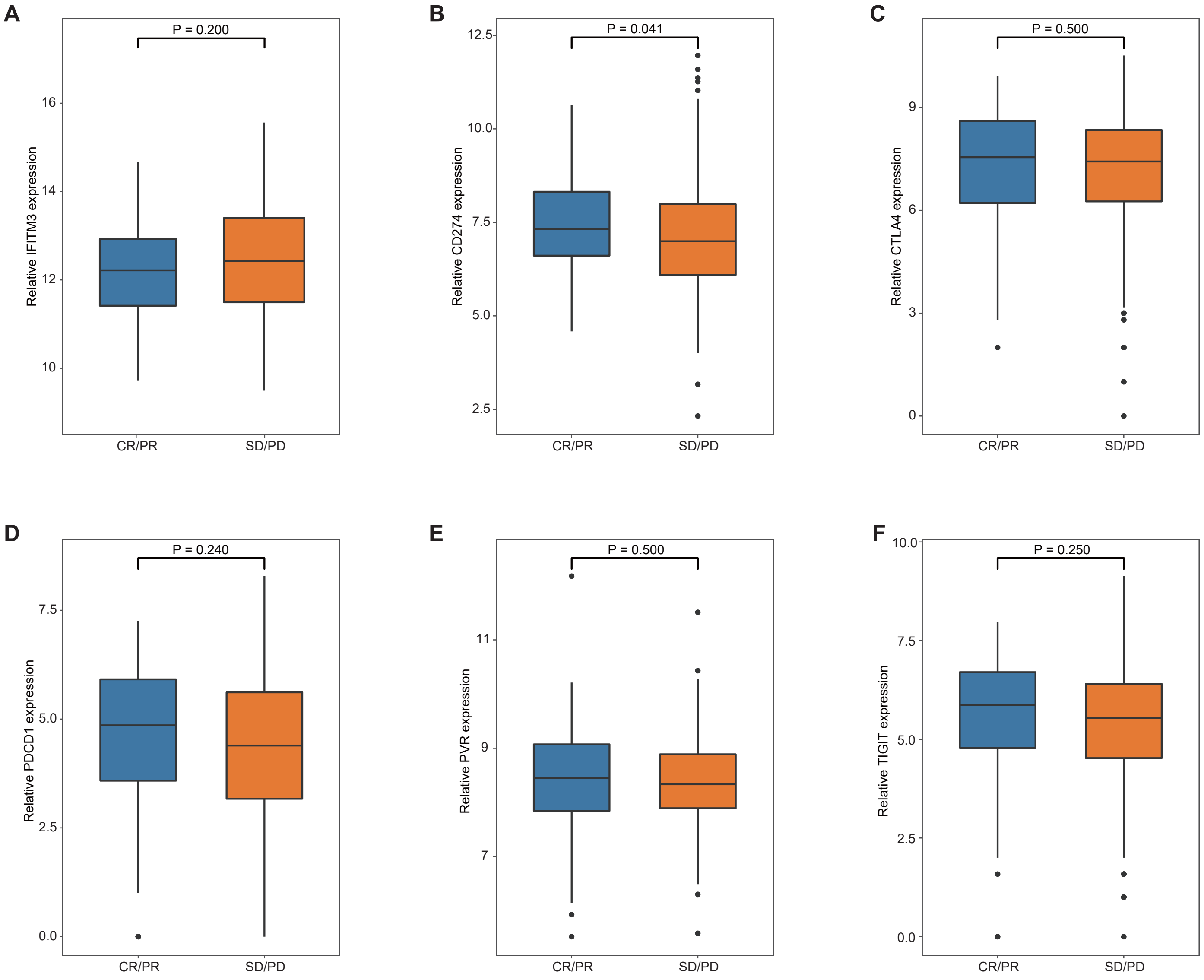

A
P = 0.200
Relative IFITM3 expression
CR/PR
SD/PD
B
P = 0.041
Relative CD274 expression
CR/PR
SD/PD
C
P = 0.500
Relative CTLA4 expression
CR/PR
SD/PD
D
P = 0.240
Relative PDCD1 expression
CR/PR
SD/PD
E
P = 0.500
Relative PVR expression
CR/PR
SD/PD
F
P = 0.250
Relative TIGIT expression
CR/PR
SD/PD

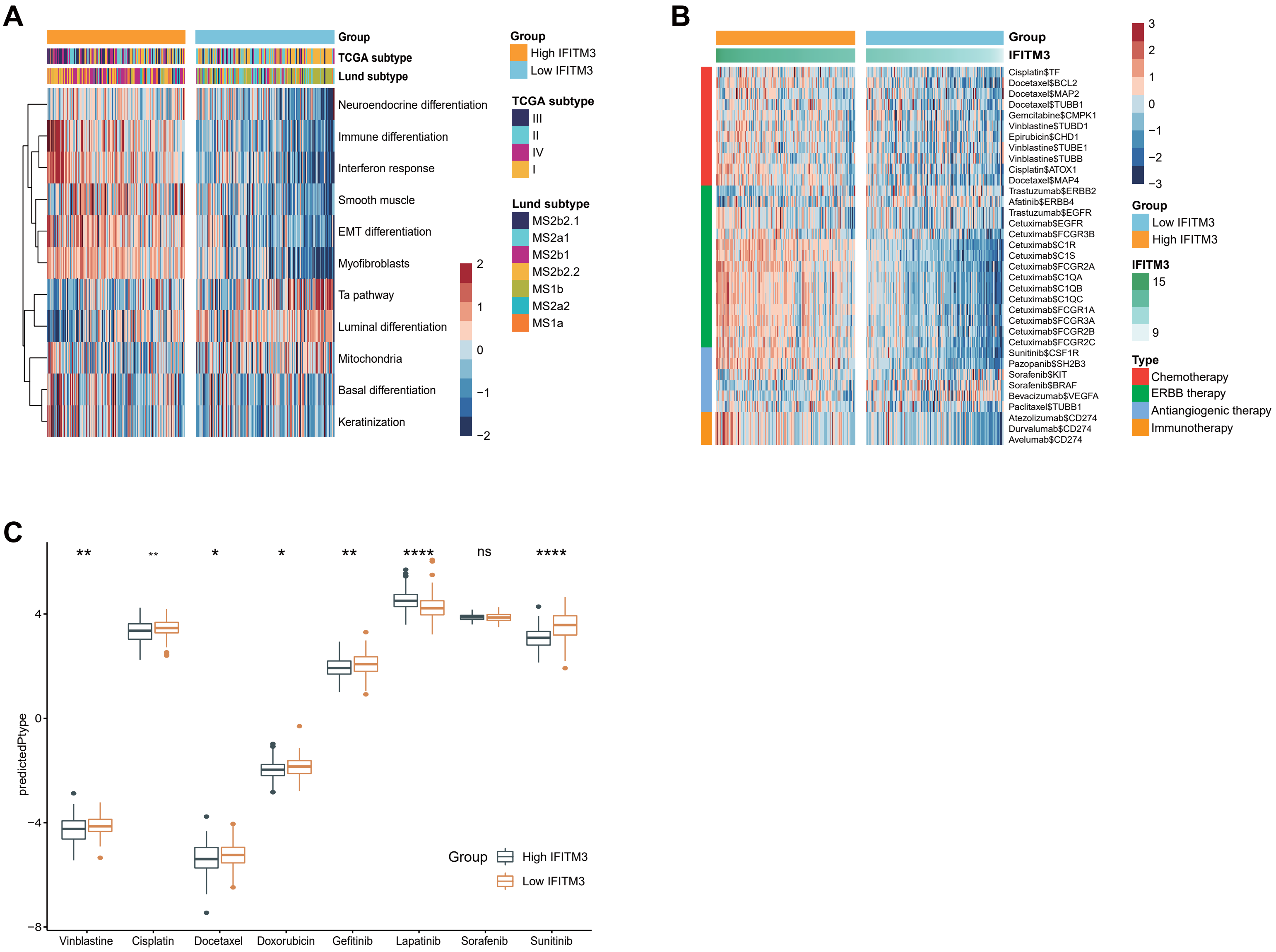

A
Group
TCGA subtype
Lund subtype
Neuroendocrine differentiation
Immune differentiation
Interferon response
Smooth muscle
EMT differentiation
Myofibroblasts
Ta pathway
Luminal differentiation
Mitochondria
Basal differentiation
Keratinization
2
1
0
−1
−2
Group
High IFITM3
Low IFITM3
TCGA subtype
III
II
IV
I
Lund subtype
MS2b2.1
MS2a1
MS2b1
MS2b2.2
MS1b
MS2a2
MS1a
B
Group
IFITM3
Cisplatin$TF
Docetaxel$BCL2
Docetaxel$MAP2
Docetaxel$TUBB1
Gemcitabine$CMPK1
Vinblastine$TUBD1
Epirubicin$CHD1
Vinblastine$TUBE1
Vinblastine$TUBB
Cisplatin$ATOX1
Docetaxel$MAP4
Trastuzumab$ERBB2
Afatinib$ERBB4
Trastuzumab$EGFR
Cetuximab$EGFR
Cetuximab$FCGR3B
Cetuximab$C1R
Cetuximab$C1S
Cetuximab$FCGR2A
Cetuximab$C1QA
Cetuximab$C1QB
Cetuximab$C1QC
Cetuximab$FCGR1A
Cetuximab$FCGR3A
Cetuximab$FCGR2B
Cetuximab$FCGR2C
Sunitinib$CSF1R
Pazopanib$SH2B3
Sorafenib$KIT
Sorafenib$BRAF
Bevacizumab$VEGFA
Paclitaxel$TUBB1
Atezolizumab$CD274
Durvalumab$CD274
Avelumab$CD274
3
2
1
0
−1
−2
−3
Group
Low IFITM3
High IFITM3
IFITM3
15
9
Type
Chemotherapy
ERBB therapy
Antiangiogenic therapy
Immunotherapy
C
predictedPtype
** ** * * ** **** ns ****
4
0
−4
−8
Vinblastine
Cisplatin
Docetaxel
Doxorubicin
Gefitinib
Lapatinib
Sorafenib
Sunitinib
Group
High IFITM3
Low IFITM3

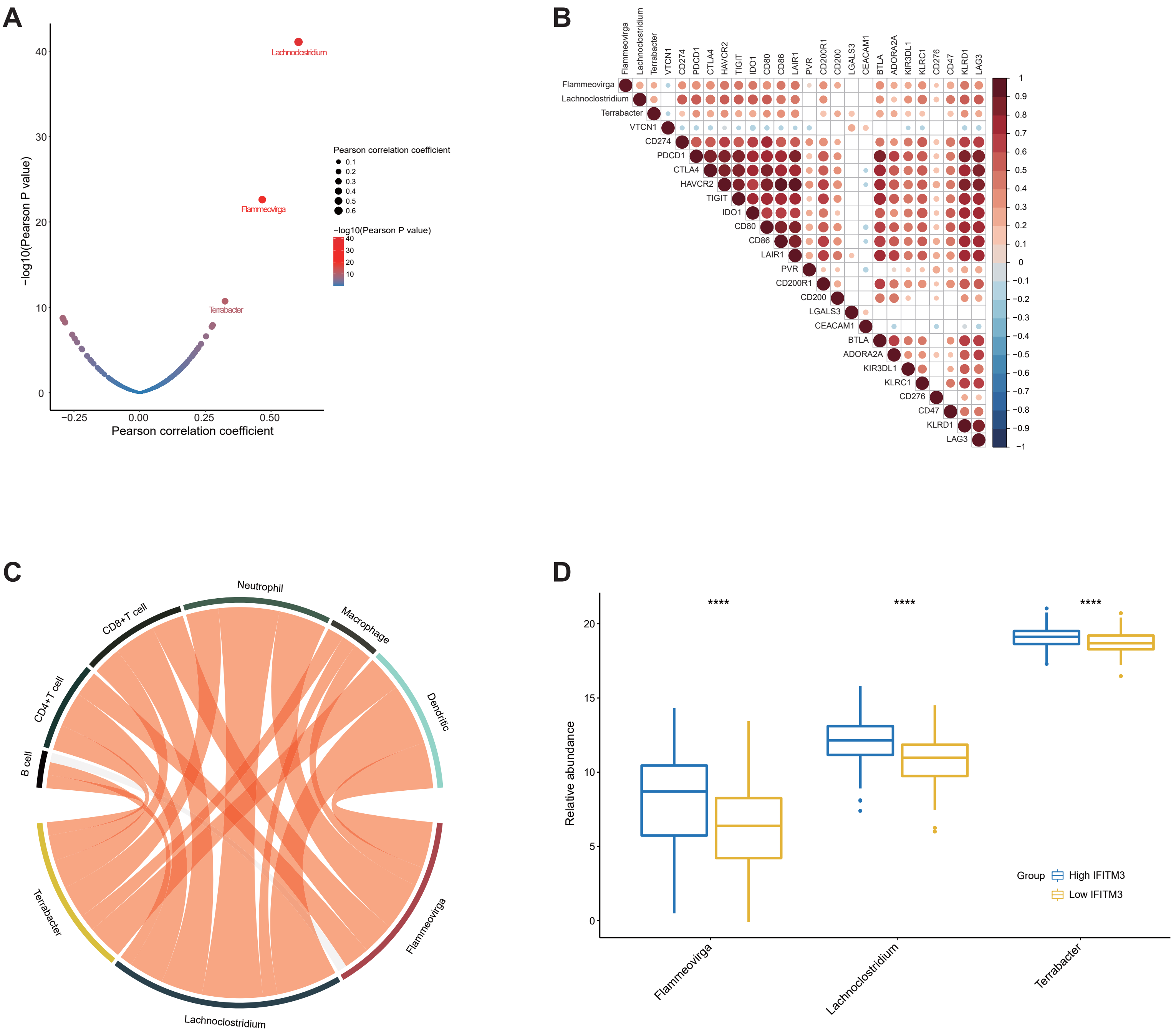

A
Lachnoclostridium
Flammeovirga
Terrabacter
−log10(Pearson P value)
Pearson correlation coefficient
Pearson correlation coefficient
−log10(Pearson P value)
B
Flammeovirga
Lachnoclostridium
Terrabacter
VTCN1
CD274
PDCD1
CTLA4
HAVCR2
TIGIT
IDO1
CD80
CD86
LAIR1
PVR
CD200R1
CD200
LGALS3
CEACAM1
BTLA
ADORA2A
KIR3DL1
KLRC1
CD276
CD47
KLRD1
LAG3
C
Neutrophil
Macrophage
Dendritic
Flammeovirga
Lachnoclostridium
Terrabacter
B cell
CD4+T cell
CD8+T cell
D
Relative abundance
Group
High IFITM3
Low IFITM3
Flammeovirga
Lachnoclostridium
Terrabacter

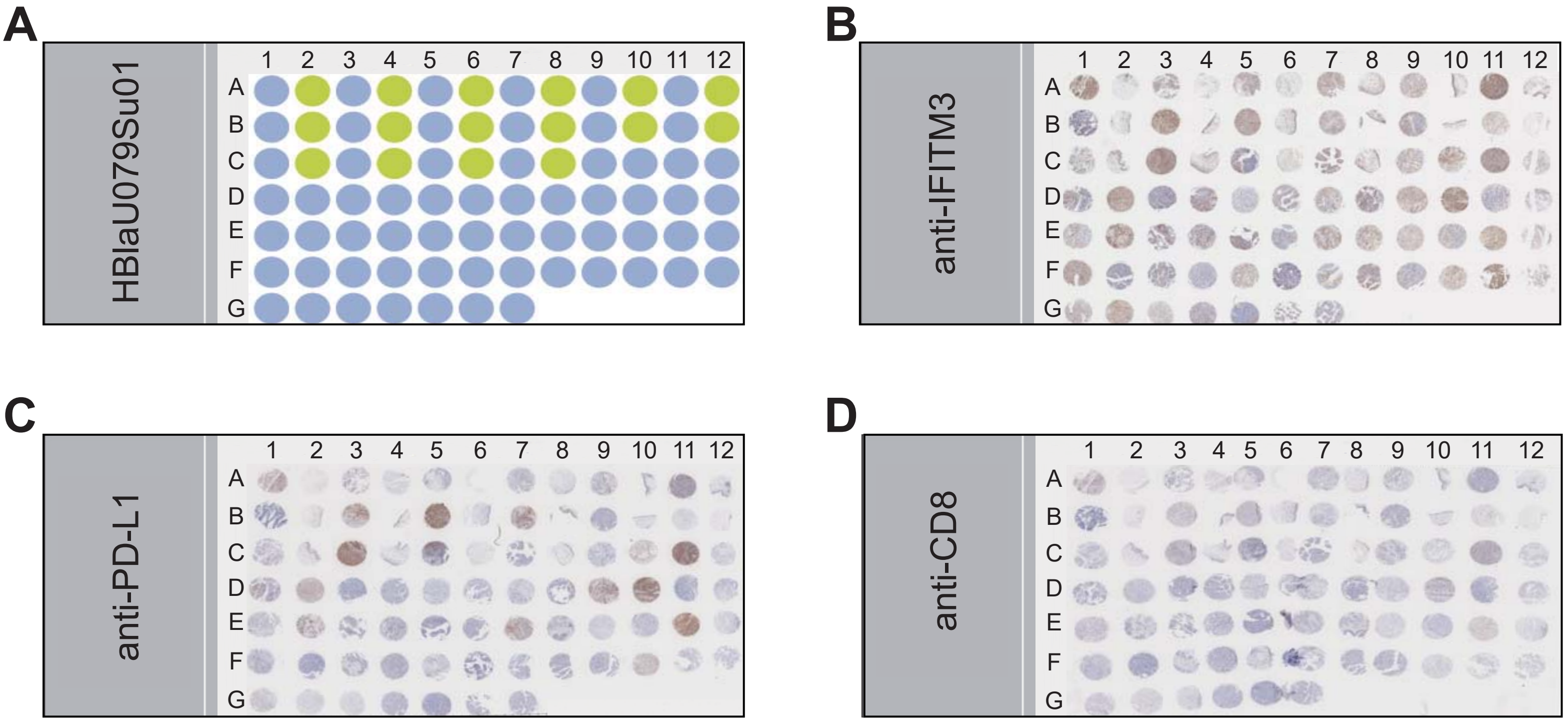
A
HBlaU079Su01
1 2 3 4 5 6 7 8 9 10 11 12
A B C D E F G
B
anti-IFITM3
1 2 3 4 5 6 7 8 9 10 11 12
A B C D E F G
C
anti-PD-L1
1 2 3 4 5 6 7 8 9 10 11 12
A B C D E F G
D
anti-CD8
1 2 3 4 5 6 7 8 9 10 11 12
A B C D E F G

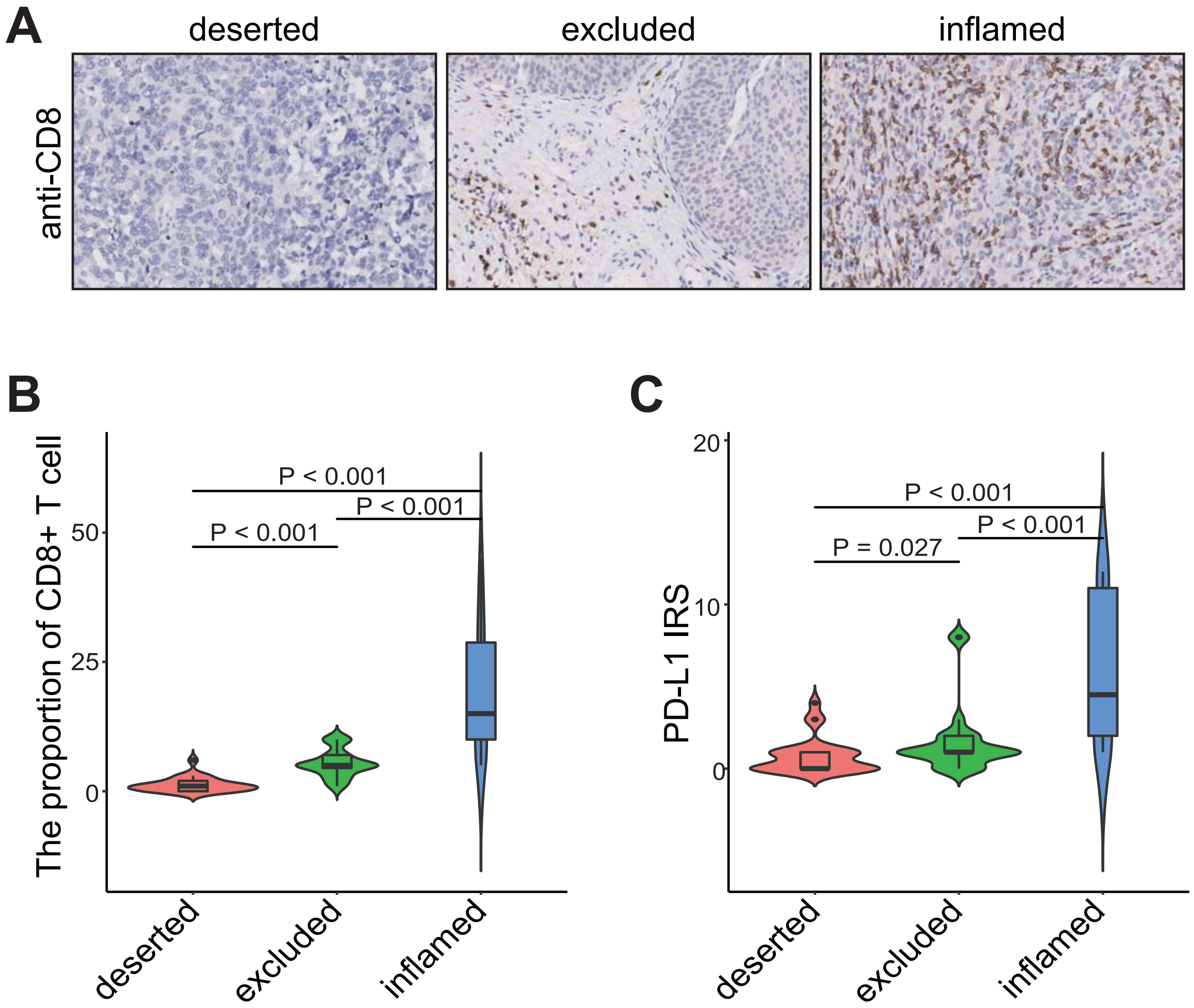
A
deserted
excluded
inflamed
anti-CD8
B
The proportion of CD8+ T cell
P < 0.001
P < 0.001
P < 0.001
50
25
0
deserted
excluded
inflamed
C
PD-L1 IRS
P < 0.001
P < 0.001
P = 0.027
20
10
0
deserted
excluded
inflamed

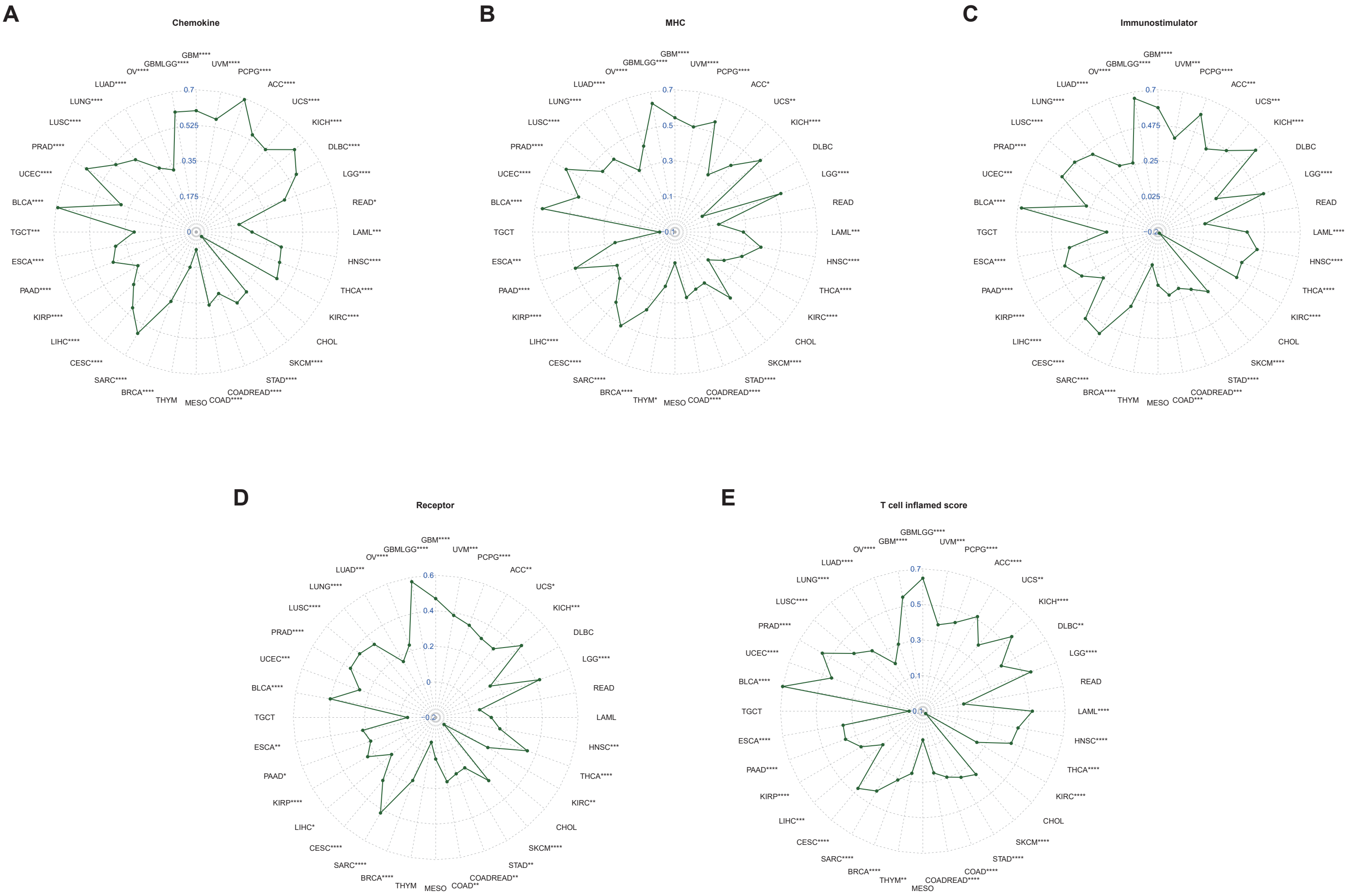
A
Chemokine
B
MHC
C
Immunostimulator
D
Receptor
E
T cell inflamed score

Table S1. Table of abbreviations.

Abbreviation	Full name
ACC	Adrenocortical carcinoma
BLCA	Bladder urothelial carcinoma
BRCA	Breast invasive carcinoma
CESC	Cervical squamous cell carcinoma and endocervical adenocarcinoma
CHOL	Cholangio carcinoma
COAD	Colon adenocarcinoma
DLBC	Lymphoid neoplasm diffuse large B-cell lymphoma
ESCA	Esophageal carcinoma
GBM	Glioblastoma multiforme
HNSC	Head and neck squamous cell carcinoma
KICH	Kidney chromophobe carcinoma
KIRC	Kidney renal clear cell carcinoma
KIRP	Kidney renal papillary cell carcinoma
LAML	Acute myeloid leukemia
LGG	Brain lower grade glioma
LIHC	Liver hepatocellular carcinoma
LUAD	Lung adenocarcinoma
LUSC	Lung squamous cell carcinoma
MESO	Mesothelioma
OV	Ovarian serous cystadenocarcinoma
PAAD	Pancreatic adenocarcinoma
PCPG	Pheochromocytoma and paraganglioma
PRAD	Prostate adenocarcinoma
READ	Rectum adenocarcinoma
SARC	Sarcoma
SKCM	Skin cutaneous melanoma
STAD	Stomach adenocarcinoma
TGCT	Testicular germ cell tumors
THCA	Thyroid carcinoma
THYM	Thymoma
UCEC	Uterine corpus endometrial carcinoma
UCS	Uterine carcinosarcoma
UVM	Uveal melanoma

Table S2. Gene Ontology (GO) and Kyoto Encyclopedia of Genes and Genomes (KEGG) pathway enrichment analyses (LinkedOmics).

Gene Set	Description	ES	NES	P value
Biological process				
GO:0002250	adaptive immune response	0.748	2.110	<0.001
GO:0007159	leukocyte cell-cell adhesion	0.728	2.039	<0.001
GO:0002449	lymphocyte mediated immunity	0.731	2.033	<0.001
GO:0032612	interleukin-1 production	0.771	2.022	<0.001
GO:0001906	cell killing	0.733	2.007	<0.001
Cell component				
GO:0005581	collagen trimer	0.765	1.993	<0.001
GO:0031012	extracellular matrix	0.696	1.971	<0.001
GO:0042611	MHC protein complex	0.913	1.930	<0.001
GO:0098552	side of membrane	0.679	1.925	<0.001
GO:0072562	blood microparticle	0.721	1.914	<0.001
Molecular function				
GO:0003823	antigen binding	0.837	2.071	<0.001
GO:0005201	extracellular matrix structural constituent	0.739	2.002	<0.001
GO:0019955	cytokine binding	0.749	1.996	<0.001
GO:0004896	cytokine receptor activity	0.757	1.958	<0.001
GO:0005539	glycosaminoglycan binding	0.672	1.867	<0.001
KEGG				
hsa05150	Staphylococcus aureus infection	0.894	2.212	<0.001
hsa04640	Hematopoietic cell lineage	0.808	2.123	<0.001
hsa04612	Antigen processing and presentation	0.823	2.089	<0.001
hsa04380	Osteoclast differentiation	0.780	2.078	<0.001
hsa04145	Phagosome	0.761	2.076	<0.001

ES: Enrichment score; NES: Normalized enrichment score.

Table S3. Correlation between T cell inflamed score and m6A genes.

m6A genes	coefficient	P value
GATA3	-0.405	<0.001
MZF1	-0.378	<0.001
METTL3	-0.364	<0.001
YTHDC1	-0.354	<0.001
YTHDF2	-0.273	<0.001
RBM15B	-0.267	<0.001
SP1	-0.262	<0.001
USP7	-0.248	<0.001
IKBKB	-0.212	<0.001
YTHDF1	-0.187	<0.001
ZMYM1	-0.184	<0.001
RBMX	-0.166	0.001
TP53	-0.132	0.008
YTHDC2	-0.128	0.010
KEAP1	-0.128	0.010
NANOG	-0.124	0.012
SOX2	-0.111	0.025
YTHDF3	-0.101	0.041
BRD4	-0.101	0.041
ZC3H13	-0.089	0.073
KIAA1429	-0.057	0.251
AFF4	-0.046	0.354
MYB	-0.013	0.801
EIF3A	0.005	0.915
HNRNPA2B1	0.008	0.870
TAZ	0.023	0.642
NOTCH1	0.028	0.570
SOCS2	0.029	0.555
SEC62	0.034	0.488
RELA	0.040	0.419
FTO	0.051	0.300
PHLPP2	0.066	0.181
SP2	0.083	0.095
PTEN	0.092	0.063
CTNNB1	0.094	0.059
HNRNPC	0.098	0.048
UBE2C	0.104	0.036
METTL14	0.105	0.034
E2F1	0.111	0.025
ALKBH5	0.113	0.023
ITGA6	0.122	0.014
IGF2BP1	0.150	0.002

FSCN1	0.215	<0.001
FOXM1	0.255	<0.001
TK1	0.256	<0.001
HDGF	0.262	<0.001
BCL2	0.264	<0.001
SRF	0.264	<0.001
WTAP	0.269	<0.001
CDCP1	0.302	<0.001
LEF1	0.308	<0.001
HIF1A	0.319	<0.001
IGF2BP3	0.322	<0.001
ETS1	0.399	<0.001
IGF2BP2	0.510	<0.001
ASB2	0.572	<0.001
CXCR4	0.603	<0.001
ADAM19	0.608	<0.001
PDCD1	0.926	<0.001
